# Association of *Fusobacterium nucleatum* with colorectal cancer molecular subtypes and its outcome: a systematic review

**DOI:** 10.1017/gmb.2025.3

**Published:** 2025-04-08

**Authors:** Luana Greco, Federica Rubbino, Clarissa Ferrari, Michela Cameletti, Fabio Grizzi, Fabrizio Bonelli, Alberto Malesci, Massimiliano Mazzone, Luigi Ricciardiello, Luigi Laghi

**Affiliations:** 1 Laboratory of Molecular Gastroenterology, IRCCS Humanitas Research Hospital, Milan, Italy; 2 Research and Clinical Trials Office, Fondazione Poliambulanza Istituto Ospedaliero, Brescia, Italy; 3 Department of Economics, University of Bergamo, Bergamo, Italy; 4 Department of Biomedical Sciences, Humanitas University, Milan, Italy; 5 Department of Immunology and Inflammation, IRCCS Humanitas Research Hospital, Milan, Italy; 6 DiaSorin SpA, Saluggia, Italy; 7 Università Vita-Salute San Raffaele, Milan, Italy; 8 Macrophage Dynamics Lab, IRCCS Humanitas Research Hospital, Milan, Italy; 9 Laboratory of Tumor Inflammation and Angiogenesis, Center for Cancer Biology, VIB, Leuven, Belgium; 10 Laboratory of Tumor Inflammation and Angiogenesis, Center for Cancer Biology, Department of Oncology, KU Leuven, Leuven, Belgium; 11 Department of Gastroenterology, Hepatology and Nutrition, The University of Texas at MD Anderson Cancer Center, Houston, TX, USA; 12 Department of Medicine and Surgery, University of Parma, Parma, Italy

**Keywords:** colorectal cancer, *Fusobacterium nucleatum*, microbiome, prognosis

## Abstract

Colorectal cancer (CRC) represents a relevant public health problem, with high incidence and mortality in Western countries. CRC can occur as sporadic (65%–75%), common familial (25%), or as a consequence of an inherited predisposition (up to 10%). While unravelling its genetic basis has been a long trip leading to relevant clinical implementation over more than 30 years, other contributing factors remain to be clarified. Among these, micro-organisms have emerged as critical players in the development and progression of the disease, as well as for CRC treatment response. *Fusobacterium nucleatum* (*Fn*) has been associated with CRC development in both pre-clinical models and clinical settings. *Fusobacteria* are core members of the human oral microbiome, while being less prevalent in the healthy gut, prompting questions about their localization in CRC and its precursor lesions. This review aims to critically discuss the evidence connecting *Fn* with CRC pathogenesis, its molecular subtypes and clinical outcomes.

## Graphical abstract

**Figure:**
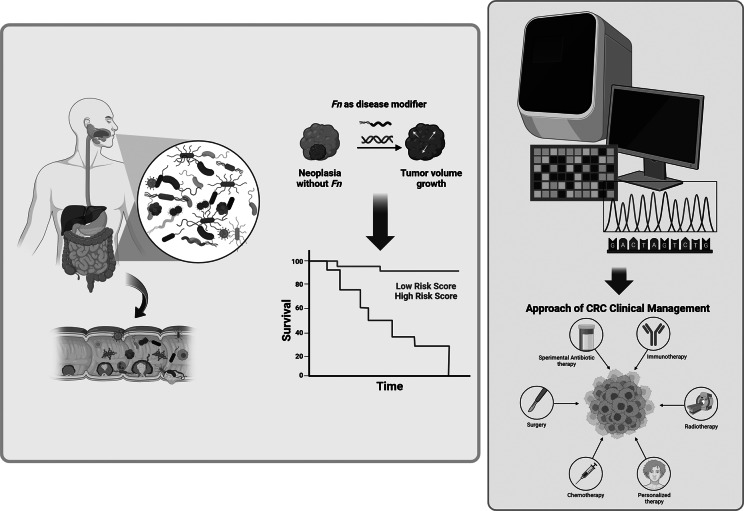
Created with BioRender.com – Last accessed online on 18 July 2024.

## Introduction

Globally, over 2.0 million new colorectal cancer (CRC) cases and 957,147 deaths are estimated to occur in 2025. Among cancers, CRC ranks second both for incidence and for mortality, despite progressive adoption of screening approaches. CRC incidence rates are about 3-fold higher in transitioned versus transitioning countries, although average case fatality remains higher in lower Human Development Index settings (Bray et al., 2018). However, even in Western Countries, up to 20%–30% of cases continue to receive clinical attention only after the onset of symptoms (Runkel et al., 2005; Decker et al., 2020), underlining a suboptimal adherence to screening programmes. Intriguingly, CRC, as a multifactorial disease, is associated with a range of individual and environmental risk factors, including patient demographics, genetic background, and lifestyle, all of which variably contribute to the risk of disease development (Robertson & Ladabaum, 2019).

## Genomic landscape of colorectal cancer

Over the past three decades, molecular genetics allowed the dissecting of the main pathways of gene damage implicated in CRC development. Eventually, the advent of next-generation sequencing (NGS) further differentiated CRC into molecular subsets. According to the pre-NGS classification, three main CRC subtypes with different clinical behaviour were defined: chromosomal instability (CIN), microsatellite instability (MSI), and the CpG island methylator phenotype (CIMP). Two major types of genomic instability were initially recognized as alternative mechanisms of CRC carcinogenesis: CIN, which occurs in 80–85% of CRCs (Grady & Carethers, 2008) and accounts for “the suppressor pathway,” characterized by aneuploidy, chromosome amplifications, and deletions (Vogelstein et al., 1989; Peinado et al., 1992; Smith et al., 2002). The gatekeeper of this pathway is the *APC* gene, which becomes inactivated according to the classic two hits model, through double somatic events in sporadic cases, or moving by a transmissible germline pathogenic variant in inherited cases in familial adenomatous polyposis coli (i.e. FAP) (Yan et al., 2002). In contrast, MSI CRCs are characterized by defects of the DNA mismatch repair (MMR) system, which lead to the progressive accumulation of unrepaired mutations (Walther et al., 2009). These tumours stay on their own under many respects. As to pathogenesis, approximately 20% arise in the context of Lynch syndrome, an inherited autosomal dominant syndrome due to germ-line mutations in one of the MMR genes (i.e. *MLH1*, *MSH2*, *MSH6*, *PMS2*, and *EPCAM*) (Laghi et al., 2008; Dal Buono et al., 2021). The remaining fraction of sporadic MSI CRCs arises through the somatic inactivation of MMR genes, most commonly via *MLH1* promoter hypermethylation, thereby accounting for a subset of the cases with the CIMP phenotype (Boland et al., 1998). Phenotypically, they have a preferential proximal location and are marked by a robust immune response. Clinically, their low metastatic potential (Malesci et al., 2007) is coupled with variable responsiveness to chemotherapy but unique responsiveness to immunotherapy, namely to PD-L1 blockade, both in adjuvant and neo-adjuvant settings (Greco et al., 2023). Accordingly, they are the first type of solid cancer amenable to non-surgical treatment with curative intent (Taieb et al., 2022).

As to precursor lesions, while Microsatellite-Stable (MSS)/CIMP-negative CRC develops through the classical adenoma-carcinoma sequence, MSI and CIMP CRCs may arise through the alternative “serrated pathway” (Jass, 2007; Kriegl et al., 2012), preferentially taking place in the right colon (Gaiser et al., 2013).

With the advent of NGS, molecular classifications evolved to identify gene expression signatures that can help stratify tumours based on their outcome and responsiveness to treatment. An approach trying to facilitate clinical translation eventually resolved initial classification inconsistencies, leading to a consensus on four main expression signaturesalso known as consensus molecular subtypes (i.e. CMS1 through CMS4). CMS1 features MSI and elicits a strong immune response. CMS2 and CMS4 show high levels of CIN, while CMS3 also includes a fraction of hypermutated tumours. CMS2, or the canonical subtype, displays epithelial features and classic WNT pathway activation. CMS3 also again has epithelial features but is notably defined by marked metabolism alterations. In contrast, CMS4 distinguished by mesenchymal and stromal infiltration signatures, altogether TGF-β activation, and downregulation of the miR-200 family (Guinney et al., 2015). The latter subtype was also identified by a different approach focused on cancer-cell intrinsic transcriptional features (or CRIS) where it was termed CRIS-B (Cancer Genome Atlas Network, 2012; Guinney et al., 2015; Isella et al., 2017).

Notwithstanding, germline mutations are increasingly reported in CRC patients following the widespread use of NGS, approximately 10% of unselected patients (Yurgelun et al., 2017), and up to 16% of juvenile (i.e. younger than 50 years of age) cases (Pearlman et al., 2017; Poliani et al., 2022). An additional 25% of cases have a positive family history with no clearly identifiable pathogenic variants and are referred to as common familial CRC, which likely recognizes polygenic bases (Kastrinos et al., 2020). Altogether, genetic predispositions and increased CRC risks associated with demographics and lifestyle call for a personalization of the screening approach (Jeon et al., 2018; Robertson & Ladabaum, 2019).

Besides the above-mentioned differences, growing evidence suggests that intestinal microbiota also plays a role in the onset and progression of CRC. The human intestinal microbiome comprises a complex community of bacteria, archaea, viruses, and eukaryotes specific to each individual and stable in healthy individuals (Song et al., 2020; Tiamani et al., 2022). Within such complex community, an increasing number of studies has shown that bacteria, chiefly *Fusobacterium nucleatum* (*Fn*), may contribute to CRC development through multiple mechanisms, including the interaction with host immune system, the production of cancer-associated metabolites, as well as the release of genotoxic virulence factors (Gholizadeh et al., 2017; Alexander et al., 2018; Hashemi Goradel et al., 2019; Justesen et al., 2022; Genua et al., 2023; Kajihara et al., 2023; Kong et al., 2023; Robinson & Allen-Vercoe, 2023; Russo et al., 2023). These interactions may also result in specific molecular signatures of CRC (Pleguezuelos-Manzano et al., 2020). The association of *Fn* with CRC is currently supported by translational evidence, and its pro-tumourigenic role is addressed by experimental models (Kostic et al., 2013; Nakatsu et al., 2015).

## Discover *Fn* in neoplastic lesions of the colon

The seminal reports concerning the enrichment of *Fusobacterium* genome in CRC lesions appeared as companion papers in *Genome Research* in 2012. The two papers are similar in their approach, results, and conclusions. The study by Castellarin *et al.* used RNA-sequencing (after host genome subtraction) to detect an over-representation of *Fn* sequences in CRC tissues as compared to matched-normal mucosa samples. Thereafter, *Fn* overabundance in CRC was confirmed by quantitative PCR analysis (Castellarin et al., 2012). The other paper, by Kostic *et al.*, used whole-genome sequencing followed by a computational subtraction to identify microbial genomes, which detected *Fn* as the enriched metagenome along with a reduced identification of *Bacteroidetes* and *Firmicutes* phyla. Amplification of *16S* rDNA and pyrosequencing confirmed the higher load of *Fn* sequences in tumour specimens, as did *in situ* hybridization (Kostic et al., 2012). Both studies also identified a positive association with lymph node metastasis and with the persistence of *Fn* sequences in distant metastases (in one-fifth of the cases) (Castellarin et al., 2012; Kostic et al., 2012).

In a later paper, Kostic *et al.* confirmed the enrichment of Fusobacteria in CRC as well as in adenomas and stool samples from patients with colonic neoplastic lesions. In parallel, they showed that *Fn* potentiates gut tumour formation in a mouse model made prone to bowel tumourigenesis through *APC* engineering, confirming the increase of *Fn* load in tumour specimens. *Fn* promotes tumour development by recruiting myeloid-derived suppressor cells (MDSCs), while simultaneously inhibiting T-cell proliferation and inducing their apoptosis within the tumour microenvironment. MDSCs express galectin-9, which binds to T-cell immunoglobulin and mucin domain-containing protein 3 (TIM-3) on lymphocytes, leading to T-cell apoptosis (Dong et al., 2002; Kostic et al., 2013). Interestingly, the metabolism of gut microbiota drives the transformation of intestinal cells not only in *APC*-derived models but also in MMR-deficient models (namely *MSH2*-deficient) (Belcheva et al., 2014).

The replication of Castellarin’s work later took place within a broader context of reproducibility in oncological research, yielding negative results (Repass et al., 2016; Repass & Reproducibility Project: Cancer Biology, 2018). The identification of microbial genomes comes with obvious challenges, including the methodology, the targeted nucleic acids, and their quantification. In tumour specimens, variability sources embrace the stage and molecular subtypes, which may be associated with different rates of positive findings. The detection of *Fn* is technically challenging and involves multiple molecular targets, including *Fn* 16S ribosomal-RNA (*rRNA*), *NusG*, *FadA, rpoB*, and methodological approaches, including amplicon sequencing, RNA sequencing, quantitative PCR, and in situ hybridization, up to the direct recovery of *Fn* from cancerous lesions. For proper comparison, we provide a list of the employed molecular targets by different PCR approaches and sequencing in Table 1.

**Table 1.** Primers and probes for quantification of Fusobacterium nucleatum DNA

Primer and probe	Sequence (5’ – 3’)	Target	Method	Reporting data	Samples analyzed	Source
*Fn*-F	GGATTTATTGGGCGTAAAGC	*16S rRNA*	qPCR	As described in Kostic et al. (2012)	Paired adenoma tissues versus adjacent normal tissues	Kostic et al. (2013)
*Fn*-R	GGCATTCCTACAAATATCTACGAA					
*Fn*-F	CAACCATTACTTTAACTCTACCATGTTCA	*16S rRNA*	Sequencing and qPCR	The fold difference (2^−ΔΔCt^) in Fusobacterium abundance between tumour and normal tissue was calculated by subtracting ΔCttumour from ΔCtnormal. The cycle threshold (Ct) values for the normal samples had a Ct range of 25.5–40, while the Ct range for the tumour samples ranged from 21.4 to 40. (two-tailed ratio t-test, p = 2.52 × 10−6).	Tumors versus adjacent normal colorectal tissue specimens	Castellarin et al. (2012)
*Fn*-R	GTTGACTTTACAGAAGGAGATTATGTAAAAATC					
*Fn*-prb	FAM - GTTGACTTTACAGAAGGAGATTATGTAAAAATC					
Pan*Fn*-F	GGATTTATTGGGCGTAAAGC	*16S rRNA*	Sequencing and qPCR	Samples were stratified as “Fusobacterium high” (qPCR Fusobacterium abundance greater than 40.0, (calculated as 1.8^[ -Ct Fusobacterium - Ct Eubacteria]x1,000), or 16S sequencing Fusobacterium abundance greater than 0.25, or “Fusobacterium low” (qPCR Fusobacterium abundance less than 1.0, or 16S sequencing Fusobacterium abundance less than 0.015).	Colorectal adenocarcinomas and adjacent non-affected tissues	Kostic et al. (2012) [primer sequences from Boutaga et al., 2005)]
Pan*Fn*-R	GGCATTCCTACAAATATCTACGAA					
Pan*Fn*-prb	FAM - CTCTACACTTGTAGTTCCG-TAMRA					
Sequencing *Fn*-F V5+357	CCTACGGGAGGCAGCAG					
Sequencing *Fn*-R V3–926	CCGTCAATTCMTTTRAGT					
*Fn*-F	GAAGAAAGAGCACAAGCTGA	*FadA*	qPCR	Copies/ng DNA >1 log difference from normal tissue.	CRC and precancerous adenomas and relatives normal tissues	Rubinstein et al. (2013)
*Fn*-R	GCTTGAAGTCTTTGAGCTCT					
P-F	CTTAGGAATGAGACAGAGATG	*16S rRNA*	qPCR			
P-R	TGATGGTAACATACGAAAGG					
*Fn*-F	GGATTTATTGGGCGTAAAGC	*16S rRNA*	qPCR	Absolute quantification by gene copy number (per gram of wet weight) – Relative abundance.	Stools	Wu et al. (2013)
*Fn*-R	GGCATTCCTACAAATATCTACGAA					
Sequencing *Fn*-F V3 +341F	NNNNNNNNCCTACGGGAGGCAGCAG		Pyro-sequencing			
Sequencing *Fn*-R V3 +533R	NNNNNNNNATTACCGCGGCTGCTGG					
*Fn*-F	CAACCATTACTTTAACTCTACCATGTTCA	*16S rRNA*	qPCR	(Tahara et al., 2014): cutoff values of 0.01 and 1 (2^-ΔCt^) were used for *Fn* and Pan-fusobacterium. A High amount of Fusobacterium (FB-high): defined for cases with either high *Fn*, or Pan-fusobacterium. or both. (Ito et al., 2015): *Fn^+^* CRCs as low or high, based on the median numbers of *Fn* (25.1 × 10^−3^ [interquartile range, 2.5 × 10^−3^−186.7 × 10^−3^]).	(Tahara et al., 2014): primary colorectal cancers and normal-appearing adjacent tissues; (Ito et al., 2015): premalignant lesions and CRCs tissue	Ito et al. (2015); Tahara et al. (2014) [primer sequences from Castellarin et al., 2012)]
*Fn*-R	GTTGACTTTACAGAAGGAGATTATGTAAAAATC					
*Fn*-prb	FAM - GTTGACTTTACAGAAGGAGATTATGTAAAAATC					
Pan*Fn*-F	GGATTTATTGGGCGTAAAGC					Ito et al. (2015); Tahara et al. (2014) [primer sequences from Boutaga et al. (2005)]
Pan*Fn*-R	GGCATTCCTACAAATATCTACGAA					
Pan*Fn*-prb	FAM - CTCTACACTTGTAGTTCCG-TAMRA					
*Fn*-F	GGATTTATTGGGCGTAAAGC	*16S rRNA*	qPCR	Level of fold increase in *Fn* over grouped those subjects with undetected *Fn* in their tumour tissue. The remaining patients with detectable *Fn* in their tumour tissue were separated into tertiles of low, moderate, and high fold increase.	Colorectal cancer and adjacent normal tissues	Flanagan et al. (2014)
*Fn*-R	GGCATTCCTACAAATATCTACGAA					
Pan*Fn*-F	CAACCATTACTTTAACTCTACCATGTTCA					
Pan*Fn*-R	GTTGACTTTACAGAAGGAGATTATGTAAAAATC					
Pan*Fn*-prb	FAM-TCAGCAACTTGTCCTTCTTGATCTTTAAATGAACC					
*Fn*-F	CAACCATTACTTTAACTCTACCATGTTCA	*NusG*	qPCR	Low or high levels based on the median cut point amount of *Fn.* Cases without detectable microbes were categorized as “negative.”	Colon and rectal carcinoma tissues	Mima et al. (2015), (2016)
*Fn*-R	GTTGACTTTACAGAAGGAGATTATGTAAAAATC					
*Fn*-prb	FAM - GTTGACTTTACAGAAGGAGATTA					
*Fn*-F	CAACCATTACTTTAACTCTACCATGTTCA	*NusG*	qPCR	Tumours with any detectable *Fn* DNA were classified as *Fn^+^*, whereas all other tumours were classified as *Fn^-^.*	Colon and rectal carcinomas tissues	Hamada et al. (2018) [primer sequences from Mima et al., 2015)]
*Fn*-R	GTTGACTTTACAGAAGGAGATTATGTAAAAATC					
*Fn*-prb	FAM - GTTGACTTTACAGAAGGAGATTA					
*Fn*-F	CAACCATTACTTTAACTCTACCATGTTCA	*16S rRNA*	qPCR	Absolute quantification by gene copies/mg (Abundance).	Stools	Mira-Pascual et al. (2015)
*Fn*-R	GTTGACTTTACAGAAGGAGATTATGTAAAAATC					
Sequencing *Fn*–25F	Universal sequence		Pyro-sequencing			
Sequencing *Fn*-F 533R	Universal sequence					
*Fn*-F	ACCTAAGGGAGAAACAGAACCA	*rpoB*	qPCR	Ct values <35 were considered positive.	Colon tissues of: hyperplastic polyp; sessile serrated adenoma; traditional adenoma; colorectal cancer; low-grade dysplasia; high-grade dysplasia	Yu et al. (2016)
*Fn*-R	CCTGCCTTTAATTCATCTCCAT					
*Fn*-targeted probe, FUS664 (FITC labelled)	CTT GTA GTT CCG C(C/T) TAC CTC	*16S rRNA*	FISH	Low and high abundance of *Fn* as those cases with <5, between 5 and 20 and >20 visualized FUS664 probes per field on average, respectively.		
Sequencing *Fn*-F V4+319F	Universal sequence	*16S rRNA*	Sequencing	Low or High on the median relative abundance as the cutoff.	Colorectal cancer tissues	Wei et al. (2016)
Sequencing *Fn*-F V4+806R	Universal sequence					
*Fn*-F	CAACCATTACTTTAACTCTACCATGTTCA	*Nus G*	qPCR	Using the median number of infectious particles in tumour tissue samples, pairs of samples in which both tumour tissue and matched NT were positive *Fn* into high- and low-abundance group.	Colorectal cancer tissues	Sun et al. (2016)
*Fn*-R	GTTGACTTTACAGAAGGAGATTATGTAAAAATC					
*Fn*-F	Not reported	*coA & 1532*	Multiplex - qPCR	Positivity was defined if the net MFI values in both duplicates were >5 MFI (median fluorescence intensity) at the Luminex analysis of PCR products	Stools	Amitay et al. (2017)
*Fn*-R	Not reported					
*Fn*-prb *Fn* Nucleatum specific	Universal sequence					
P-F	AAGCGCGTCTAGGTGGTTATGT	*16S rRNA*	Digital PCR	The median copy numbers of *Fn* were 17.5 (range, 0–5793) in the control group, 311 (range, 1–5305) in the non-advanced adenoma group, 122 (range, 1–9481) in the advanced adenoma/CIS group, and 317 (range, 0–17343) in the CRC group.	Stools	Suehiro et al. (2017)
P-R	TGTAGTTCCGCTTACCTCTCCAG					
Prb	FAM - CAACGCAATACAGAGTTGAGCCCTGCATT-BHQ1					
*Fn*-F	CAACCATTACTTTAACTCTACCATGTTCA	*NusG*	qPCR	The abundance of the microbial markers was calculated as a relative unit normalized to the total bacteria using the 2^−ΔCt^ method. Best cut-off value of 1.5×10^−6^.	Stools	Wong et al. (2017)
*Fn*-R	GTTGACTTTACAGAAGGAGATTATGTAAAAATC					
*Fn*-F	CAACCATTACTTTAACTCTACCATGTTCA	*NusG*	qPCR	*Fn* positivity was defined as a detectable level of F nucleatum DNA within 45 PCR cycles, and F nucleatum negativity was defined as an undetectable level.	Colorectal cancer tissues	Mehta et al. (2017) [primer sequences from Mima et al. (2015)]
*Fn*-R	GTTGACTTTACAGAAGGAGATTATGTAAAAATC					
*Fn*-prb	FAM - GTTGACTTTACAGAAGGAGATTA					
*Fn*-F	AAGCGCGTCTAGGTGGTTATGT	*16S rRNA*	qPCR	(Bullman et al., 2017): *Fn*-high if relative abundance (RA) >1%, and *Fn* -Low/Neg if <1% Fusobacterium RA. (Yamaoka et al., 2018): *Fn* -high: *Fn* >1.9 copies/ng DNA; *Fn* -low: *Fn* ≤1.9 copies/ng DNA.	(Bullman et al., 2017): primary colorectal carcinomas and corresponding liver metastases (Yamaoka et al., 2018): colorectal cancer tissues and matched normal-appearing mucosal tissues.	Bullman et al. (2017); Yamaoka et al. (2018) [primer sequences from Martin et al., 2002)]
*Fn*-R	TGTAGTTCCGCTTACCTCTCCAG					
*Fn*-prb	FAM - CAACGCAATACAGAGTTGAGCCCTGCATT-TAMRA					
*Fn*-F	CTTAGGAATGAGACAGAGATG	*16S rRNA*	qPCR	The optimal cutoff value of *Fn* level in CRC tissues was 0.0282. *Fn* -High>0,0282; *Fn* -Low/Neg<0,0282.	Colorectal cancer and adjacent normal tissues	Yan et al. (2017)
*Fn*-R	TGATGGTAACATACGAAAGG					
P-F	GGATTTATTGGGCGTAAAGC	*16S rRNA*	qPCR	*Fn* Cut-off value = –18 of relative aboundance (Log2).	Stools	Xie et al. (2017) [primer sequences from Kostic et al., 2013)]
P-R	GGCATTCCTACAAATATCTACGAA					
*Fn*-F	CAACCATTACTTTAACTCTACCATGTTCA	*NusG*				Xie et al. (2017) [primer sequences from Wong et al., 2017)]
*Fn*-R	GTTGACTTTACAGAAGGAGATTATGTAAAAATC					
*Fn*-F	CCATGAAGTCGGAATCGCTAG	*16S rRNA*	qPCR	Youden index =0.00026 for the relative levels of *Fn* (2−^Δ Cq^) was used to identify the cutoff for *Fn^+^* (with high levels of *Fn*) or *Fn^-^* (with low levels of *Fn*).	Stools	Eklöf et al. (2017)
*Fn*-R	GCTTGACGGGCGGTGT					
P-F	CGTCAGCTCGTGYCGTGAG	*16S rRNA*	qPCR	Relative abundance of *Fn.* The best cut-off values were determined by ROC analyses that maximized the Youden index (J = Sensitivity + Specificity − 1) = 0.0007072.	Stools	Liang et al. (2021); Liang et al. (2017)
P-R	CGTCRTCCCCRCCTTCC					
Prb	VIC-TTAAGTCCCRYAACGAGCGCAACCC-TAMRA					
*Fn*-F	TTCAATAAAAGTGGCAGGTCAAG	*NusG*		Relative abundance was calculated by using the delta Cq method as compared with internal control. The best cut-off *Fn* values were determined by ROC analyses that maximized the Youden index (J = Sensitivity + Specificity − 1) = 7.43.		
*Fn*-R	TAACAACACATGCAGGTCAATGG					
*Fn*-prb	FAM - ACTCGAACCCCCAACCCTCGGTTT-TAMRA					
*Fn*-F	CAACCATTACTTTAACTCTACCATGTTCA	*16S rRNA*	Sequencing and qPCR	Results reported as fold difference (2^−ΔΔCt^) in Fusobacterium abundance were calculated by subtracting ΔCt tumour from ΔCt normal. Ct values were 22.4 to 40 for adjacent normal tissue, 22.1 to 40 for CRC tissue, and 21.8 to 40 for matched normal tissue.	Tumors versus normal colorectal carcinoma tissues	Repass & Reproducibility Project: Cancer Biology (2018)
*Fn*-R	GTTGACTTTACAGAAGGAGATTATGTAAAAATC					
*Fn*-prb	FAM - GTTGACTTTACAGAAGGAGATTATGTAAAAATC					
*Fn*-F	CAACCATTACTTTAACTCTACCATGTTCA	*16S rRNA*	qPCR	Cases with positive *Fn* were categorized further as low or high relative to the median cut-off point *Fn* DNA quantities among *Fn* ^+^ cases.	Colorectal cancer tissues	Liu et al. (2018) [primer sequences from Castellarin et al. (2012)]
*Fn*-R	GTTGACTTTACAGAAGGAGATTATGTAAAAATC					
*Fn*-prb	FAM - GTTGACTTTACAGAAGGAGATTATGTAAAAATC					
*Fn*-F	CAACCATTACTTTAACTCTACCATGTTCA	*NusG*	qPCR			Liu et al. (2018) [primer sequences from Mima et al., 2015)]
*Fn*-R	GTTGACTTTACAGAAGGAGATTATGTAAAAATC					
*Fn*-prb	FAM - GTTGACTTTACAGAAGGAGATTA					
*Fn*-F	CAACCATTACTTTAACTCTACCATGTTCA	*NusG*	qPCR	High levels of *Fn* were defined using ∆Ct (Ct *Fn* − Ct16S rRNA), setting a cutoff of 12.	Stools and colonic tissues	Tunsjø et al. (2019)
*Fn*-R	TACTGAGGGAGATTATGTAAAAATC					
*Fn*-F	CAACCATTACTTTAACTCTACCATGTTCA	*16S rRNA*	qPCR	*Fn^+^* CRCs were further classified into two subgroups (*Fn-*high or *Fn-*low) using a cut-off median value of 2^-ΔCt^	Primary colorectal cancer tissues	Oh et al. (2019)
*Fn*-R	GTTGACTTTACAGAAGGAGATTATGTAAAAATC					
*Fn*-prb	FAM-GTTGACTTTACAGAAGGAGATTA					
*Fn*-F	GATCCAGCAATTCTGTGTGC	*NusG*	qPCR	*Fn* amount was defined as *Fn* concentration/*SLCO2A1* concentration. If DNA was not detected until 40 cycles, we defined the patient’s Ct value as 40.	Colorectal cancer tissues compared with adjacent normal tissues	Lee et al. (2018)
*Fn*-R	TTGTAGTTCCGCYTACCTC					
*Fn*-prb	GGACTACCAGGGTATCTAATCCTGTT					
Sequencing *Fn*-F V4 +515F	TCGTCGGCAGCGTCAGATGTGTATAAGAGACAGCCTACGGGNGGCWGCAG	*16S rRNA*	Sequencing	Presence or absence of bacteria.	Colorectal adenocarcinoma, colorectal adenomas, and diverticular disease tissues	Bundgaard-Nielsen et al. (2019)
Sequencing *Fn*-F V4 +806R	GTCTCGTGGGCTCGGAGATGTGTATAAGAGACAGGACTACHVGGGTATCTAATCC					
*Fn*-F [locus-specific sequence]	CCCAAGCAAACGCGATAAGT					
*Fn*-R [locus-specific sequence]	GCGTTGCGTCGAATTAAACC					
*Fn*-F	CAACCATTACTTTAACTCTACCATGTTCA	*16S rRNA*	qPCR	*Fn*-high or *Fn*-low using a cut-off median value of 2^-ΔCt.	Colorectal cancer tissues	Chen et al. (2019)
*Fn*-R	GTTGACTTTACAGAAGGAGATTATGTAAAAATC					
*Fn*-prb	FAM-GTTGACTTTACAGAAGGAGATTA					
Sequencing *Fn*-F V4 +515F	GTGCCAGCMGCCGCGGTAA	*16S rRNA*	Sequencing	According to the amount of Bacteria found as low or high (*Fn*-low and *Fn*-high), on the basis of the median cut point amount of *Fn* DNA in all samples with positive results (tumour or normal adjacent).	Colorectal cancer and adjacent normal tissues	de Carvalho et al. (2019)
Sequencing *Fn*-F V4 +806R	GGACTACHVGGGTWTCTAAT					
*Fn*-F	CAACCATTACTTTAACTCTACCATGTTCA	*Nus G*	qPCR			
*Fn*-R	GTTGACTTTACAGAAGGAGATTATGTAAAAATC					
*Fn*-prb	FAM-GTTGACTTTACAGAAGGAGATTA-MGBNFQ					
*Fn*-F	CAACCATTACTTTA ACTCTACCATGTTCA	*Nus G*	qPCR	*Fn* abundance (in individuals with the bacterium detected) was divided into low and high groups based on values below or above the median value, respectively.	Colorectal cancer and adjacent normal tissues	Kunzmann et al. (2019)
*Fn*-R	GTTGACTTTACAGAAGGAGATTATGTAAAAATC					
*Fn*-prb	FAM-CAGCAACTT GTCCTTCTTGATCTTTAAATGAACC					
Sequencing *Fn*-F V3 +341F (Illumina 5’ sequencing adapters + V3-specific primers)	ACACTGACGACATGGTTCTACA + CCTACGGGGGGCAGCAG	*16S rRNA*	Sequencing	*Fn* abundance (in individuals with the bacterium detected) was divided into low and high groups based on values below or above the median value, respectively.	Colorectal cancer tissues	Leung et al. (2019)
Sequencing *Fn*-R V4 + 806R (Illumina 5’ sequencing adapters + V4-specific primers)	TACGGTAGCAGAGACTTGGTCT + GGACTACCGGGGTATCT					
*Fn*-F	AAGCGCGTCTAGGTGGTTATGT		Digital PCR			
*Fn*-R	TGTAGTTCCGCTTACCTCTCCAG					
*Fn*-prb	FAM - CAACGCAATACAGAGTTGAGCCCTGCATT-BHQ1					
*Fn*-F	CAACCATTACTTTAACTCTACCATGTTCA	*NusG*	qPCR	Tumours with any detectable *Fn* DNA were classified as *Fn^+^*, whereas all other tumours were classified as *Fn.^-^*	Colorectal cancer tissues	Haruki et al. (2020) [primer sequences from Mima et al., 2015)]
*Fn*-R	GTTGACTTTACAGAAGGAGATTATGTAAAAATC					
*Fn*-prb	FAM - GTTGACTTTACAGAAGGAGATTA					
*Fn*-F	TGGTGTCATTCTTCCAAAAATATCA	*NusG*	qPCR	Low or high on the basis of the median cut point amount of *Fn.* Cases without detectable microbes were categorized as “negative.”	Colorectal cancer tissues	Mima et al. (2020).
*Fn*-R	AGATCAAGAAGGACAAGTTGCTGAA					
*Fn*-prb	FAM - ACTTTAACTCTACCATGTTCA					
P-F	GGATTTATTGGGCGTAAAGC	*16S rRNA*				
P-R	GGCATTCCTACAAATATCTACGAA					
*Fn*-F	CGGTGGAGCATGTGGTTTAA	*16S rRNA*	qPCR	Low or High using a cut-off median value of 2−ΔCt of all *Fn^+^* cases.	Colorectal cancer tissues	Lee et al. (2021)
*Fn*-R	TTCCTAAGATGTCAAACGCTGG					
*Fn*-prb	FAM-TCGACGCAACGCGAGGAACCTT-BHQ1					
Sequencing *Fn*-F V4	Universal sequence	*16S rRNA*	Sequencing	Heatmap	Colorectal cancer tissue biopsies	Liu et al. (2021)
Sequencing *Fn*-F V4	Universal sequence					
*Fn*-F	CAACCATTACTTTAACTCTACCATGTTCA	*NusG*	qPCR	Comparative analysis in retrospective and prospective cohort. The optimal cut-off value is determined when the value reaches both the highest Youden index with the highest sensitivity in the retrospective cohort to maximize screening efficiency. The best cut-off, *Fn* relative abundance = 0.4491.	Stools	Xue et al. (2021) [primer sequences from Wong et al., 2017, p. 2)]
*Fn*-R	GTTGACTTTACAGAAGGAGATTATGTAAAAATC					
P-F	GGTGAATACGTTCCCGG	*16S rRNA*				
P-R	TACGGCTACCTTGTTACGACTT					
*Fn*-F	CAACCATTACTTTAACTCTACCATGTTCA	*NusG*	qPCR	The relative abundance of *Fn* was calculated in proportion to the total bacterial load as the ratio of 2 (−*Fn*) /2 (−*16S*) average Ct values.	Stools	Eisele et al. (2021) [primer sequences from Tunsjø et al., 2019)]
*Fn*-R	TACTGAGGGAGATTATGTAAAAATC					
SR-n1 N1-F	GAGATGGAAGTACAAAACAAATTACAGTAG	*C7Y58_07315*	Whole-genome phylogenetic analysis	Heatmap	Stools and fresh-frozen tumor tissues	Bi et al. (2021) [primer sequences from (Castellarin et al. (2012); Geller et al. (2017)]
SR-n1 N1-R	TCTTGGACATCAACATCATAAACAGA					
SR-n2 N2-F	GAGTTACAACTTCCTATATTTGACCGA	*C7Y58_06070*				
SR-n2 N2-R	CAAACATTTTGATAATTCATCTGCAT					
SR-n3 N3-F	CAAGCAACTGAAAATGCTTTAAAAG	*C7Y58_06085*				
SR-n3 N3-R	TCCAGGTAAGGAAATTACACCTACTG					
SR-n4 N4-F	GGGAGCTACATTTTTCCTACTCTTCC	*C7Y58_06080*				
SR-n4 N4-R	TCTATCTACACCTTCAGCAGCCTTT					
SR-n5 N5-F	AAACTTACTGTCTATCCCTATGCTTTAA	*C7Y58_05110*				
SR-n5 N5-R	CTTTATTCAAGTAGGGTTCTATCCATAAT					
SR-n6 N6-F	GATAGACAACGAAGAGAAAGAGAAGCT	*C7Y58_08045*				
SR-n6 N6-R	GTATCCTCATTCCATACAAAGACTGC					
Pan*Fn*	CNACGCGAAGAACCTTANC	*16S rRNA*	Sequencing	Presence or absence of bacteria.		
Pan*Fn*	ATACGCGARGAACCTTACC					
Pan*Fn*	CTAACCGANGAACCTYACC					
Pan*Fn*	CAACGCGMARAACCTTACC					
Pan*Fn*	CGACRRCCATGCANCACCT					
*Fn*-F	CAACCATTACTTTAACTCTACCATGTTCA	*16S rRNA*				
*Fn*-R	GTTGACTTTACAGAAGGAGATTATGTAAAAATC					
*Fn*-prb	FAM - GTTGACTTTACAGAAGGAGATTATGTAAAAATC					
*Fn*-F	AGAGTTTGATCCTGGCTCAG	*16S rRNA*	qPCR	Presence or absence of bacteria.	Stools	Li et al. (2021) [primer sequences from Liu et al., 2014)]
*Fn*-R	GTCATCGTGCACACAGAATTGCTG					
*Fn*-F	CACAAGCTGACGCTGCTAGA	*FadA*				
*Fn*-R	TTACCAGCTCTTAAAGCTTG					
*Fn*-F	Gene bank Acc. FJ471654.1	*16S rRNA*	qPCR	Log Abundance	CRC and precancerous-benign colon disease (P-BCD) tissues	Kurt and Yumuk (2021)
*Fn*-R						
Antibodies	Serum-specific antibody to F. nucleatum	IgA and IgG	ELISA	0.868 and 0.133 for IgA and IgG, respectively, calculated on CRC patients.		
*Fn*-F	CAACCATTACTTTAACTCTACCATGTTCA	*NusG*	qPCR	Mean quantitative polymerase chain reaction mean threshold cycles ± SD of *NusG* and *16S* rRNA Genes.	Pairs of frozen sporadic CRC tumor and adjacent non-tumor mucosal tissue	Malik et al. (2021)
*Fn*-R	GTTGACTTTACAGAAGGAGATTATGTAAAAATC					
*Fn*-prb	CAGCAACTTGTCCTTCTTGATCTTTAAATGAACC					
Pan*Fn*-F	TCCTACGGGAGGCAGCAGT	*16S rRNA*				
Pan*Fn*-R	GGACTACCAGGGTATCTAATCCTGTT					
Pan*Fn*-prb	CGTATTACCGCGGCTGCTGGCAC					
Commercial platform	Commercial platform	Sixty-seven different metabolic pathways	Shotgun Metagenomic Sequencing MiSeq™ System and in silico analysis.	Heatmap	Stool samples and NCBI database (ref_CRC, Metagenomics sequencing data: PRJEB7774)	Chang et al. (2021)
		4,071,018 microbial genes obtained by MetaGeneMark (version 3.26) and CD-HIT (version 4.5.7)	Shotgun Metagenomic Sequencing MiSeq™ System, Illumina	Heatmap	Stool samples of CRC, adenomas and Healthy subjects	Gao et al. (2021)
*Fn*-F	CAACCATTACTTTAACTCTACCATGTTCA	*NusG*	qPCR	Positivity was defined as a detectable level of bacterial DNA, and negativity was defined as an undetectable Level.	Colorectal cancer and adjacent normal tissues	Sikavi et al. (2021) [primer sequences from Mima et al. (2015)]
*Fn*-R	GTTGACTTTACAGAAGGAGATTATGTAAAAATC					
*Fn*-prb	FAM - GTTGACTTTACAGAAGGAGATTA					
*Fn*-F	CGCAGAAGGTGAAAGTCCTGTAT	*16S rRNA*	qPCR	Relative quantification and determined by 2-ΔCt, where ΔCt was the difference in the Ct number for the test and reference (Glyceraldehyde–3-phosphate dehydrogenase[*GAPDH*]) gene assay. The abundance of *Fn* determined through the 2-ΔΔCt method in CRC (7.750 [5.790–10.469]) was significantly higher than in the normal control (0.409 [0.251–0.817]) (p < 0.05).	Normal colorectal tissue, peritumor and CRC tissues	Cuellar-Gómez et al. (2022)
*Fn*-R	TGGTCCTCACTGATTCACACAGA					
Sequencing *Fn*-F V3	AATGATACGGCGACCACCGAG-TATGGTAATTGTGTGCCAGCMGCCGCGGTAA		Sequencing			
Sequencing *Fn*-R V6	CAAGCAGAAGACGGCATACGAGAT-GGACTACHVGGGTWTCTAAT					
*Fn*-F	CAACCATTACTTTAACTCTACCATGTTCA	*NusG*	qPCR	Relative quantification and determined by 2-ΔCt	Saliva, Oral rinse, Colorectal and tumor mucosa, Stools	Idrissi Janati et al. (2022) [primer sequences from Mima et al. (2015)]
*Fn*-R	GTTGACTTTACAGAAGGAGATTATGTAAAAATC					
*Fn*-prb	FAM - GTTGACTTTACAGAAGGAGATTA					
*Fn*-F	CAACCATTACTTTAACTCTACCATGTTCA	*16S rRNA*	qPCR	*Fn* positivity was defined as a detectable level of *Fn* DNA within 40 PCR cycles, and *Fn* negativity was defined as an undetectable level with a proper amplification of human reference gene *SLCO2A1* and/or *MEFE.*		Idrissi Janati et al. (2022)
*Fn*-R	GTTGACTTTACAGAAGGAGATTATGTAAAAATC					
*Fn*-prb	GTTGACTTTACAGAAGGAGATTA					
Reference gene	Ba042114926-s1, FisherThermoScientific, USA	*MEFE*				
Reference gene	Hs01114926_m1, FisherThermo Scientific, USA	*SLCO2A1*				
*Fn*-F	TGGTGTCATTCTTCCAAAAATATCA	*NusG*	qPCR	The abundance of *Fn* was calculated using the 2-ΔCt method, in which ΔCt refers to the difference in Ct number between *Fn* and the *SLCO2A1* reference gene.	Colorectal cancer tissue	Chen et al. (2022) [primer sequences from (Mima et al. (2020)]
*Fn*-R	AGATCAAGAAGGACAAGTTGCTGAA					
*Fn*-prb	FAM-ACTTTAACTCTACCATGTTCA					
HouseKeeping-F	ATCCCCAAAGCACCTGGTTT	*SLCO2A1*				
HouseKeeping-R	AGAGGCCAAGATAGTCCTGGTAA					
HouseKeeping-prb	VIC-CCATCCATGTCCTCATCTC					
*Fn*-F	CAACCATTACTTTAACTCTACCATGTTCA	*NusG*	qPCR	Samples were considered positive if two of the three replicates amplified *Fn* DNA.	Colonic biopsies and stools	Aitchison et al. (2022) [primer sequences from Flanagan et al., 2014)]
*Fn*-R	GTTGACTTTACAGAAGGAGATTATGTAAAAATC					
*Fn*-prb	TCAGCAACTTGTCCTTCTTGATCTTTAAATGAACC					
HouseKeeping-F	ATCCCCAAAGCACCTGGTTT	*PGT*				
HouseKeeping-R	AGAGGCCAAGATAGTCCTGGTAA					
HouseKeeping-prb	CCATCCATGTCCTCATCTC					
*Fn*-F	TGGTGTCATTCTTCCAAAAATATCA	*Nus-G*	qPCR	Samples were divided into low-*Fn* and high-*Fn* DNA by Youden index=(sensitivity + specificity–1) as the cutoff value of salivary *Fn* DNA was 0.437.	Saliva, serum and tumor tissue	Zhang et al. (2022) [primer sequences from (Mima et al. (2020)]
*Fn*-R	AGATCAAGAAGGACAAGTTGCTGAA					
*Fn*-prb	ACTTTAACTCTACCATGTTCA					
HouseKeeping-F	AGGTTTACATGTTCCAATATGATTCCA	*GAPDH*				
HouseKeeping-R	ATGGGATTTCCATTGATGACAAG					
HouseKeeping-prb	CCGTTCTCAGCCTTGACGGTGC					
HouseKeeping-F	CCTCACATAAATGCTACCAAACGA	*TERT*				
HouseKeeping-R	TTCCAAGAAGGAGGCCATAGTC					
HouseKeeping-prb	AAGAAATGAACAGACCCATCCCCCAGG					
Sequencing *Fn*-F V3 +341F (Illumina 5’ sequencing adapters + V3-specific primers)	ACACTGACGACATGGTTCTACA + CCTACGGGGGGCAGCAG	*16S rRNA*	Sequencing	Samples were divided in *Fn*-high and *Fn*-no cases by cut off of Gene expression score of 0,042	Primary colon cancer tissue and matched distant non-neoplastic colon tissue, Stools	Ternes et al. (2022)
Sequencing *Fn*-R V4 + 806R (Illumina 5’ sequencing adapters + V4-specific primers)	TACGGTAGCAGAGACTTGGTCT + GGACTACCGGGGTATCT					
*Fn*-F	CAACCATTACTTTAACTCTACCATGTTCA	*NusG*	qPCR	Low or high levels based on the median cut point amount of *Fn.* Cases without detectable microbes were categorized as “unknown.”	Primary colon cancer tissue	Joo et al. (2024) [primer sequences from Mima et al. (2015)]
*Fn*-R	GTTGACTTTACAGAAGGAGATTATGTAAAAATC					
*Fn*-prb	FAM - GTTGACTTTACAGAAGGAGATTA					

Despite promising findings over the last 10 years, the role of the microbiome in the natural history of CRC has not yet been clarified (Carethers & Doubeni, 2020), neither the identification of specific bacteria has been translated into clinical practice.

We reviewed the evidence supporting the association between *Fn* and CRC, with a focus on molecular subtypes and host immune response, as well its potential role as a prognostic and predictive marker. Modifiable factors, such as diet as well as potential exploitation of *Fn* as a therapeutic target, will also be covered from a translational perspective, foreseeing its potential clinical implications.

## Topography of *Fusobacterium nucleatum* in the gastrointestinal tract

*Fusobacterium nucleatum* is a Gram-negative anaerobic bacterium belonging to the *Bacteroidaceae* family. *Fusobacteria* species differ from other *Bacteroidaceae* in their ability to produce N-butyrate and modulate canonical Wnt signalling, thereby promoting tumourigenesis (Lazarova et al., 2004; Lupton, 2004; Flanagan et al., 2014). Although *Fn* is described as an obligate anaerobe, it can grow in up to 6% oxygen (Moore et al., 1984). *Fn* represents a small proportion of the commensal microflora of the mouth in humans (Kapatral et al., 2002; Brennan & Garrett, 2019), and in this milieu, it is symbiotic with other bacteria, but herein and elsewhere, it could also act as an opportunistic pathogen, having been isolated from skin ulcers, peritonsillar abscesses, septic arthritis, and endocarditis (Kapatral et al., 2002). It remains unclear whether in these infective contexts it acts like a driver or a passenger.

How *Fn* colonizes gut mucosa from the oral cavity remains debated (Mesa et al., 2022; Zhang et al., 2018). It has been reported that, although the taxonomic compositions of the oral and gut microbiomes differ, community types observed at these sites can predict each other, showing significant associations between saliva and gut specimens. These results suggest that oral bacterial populations may seed the gut, and by the time they reach the stool, those populations experience the ecological environment of the gut and give rise to consistent community types (Wu et al., 2013; Ding & Schloss, 2014; Mira-Pascual et al., 2015; Idrissi Janati et al., 2022). A rat model exploring the correlation between the development of apical periodontitis induced by *Fn* inoculation and related changes in the intestinal flora found that it negatively affects the anti-inflammatory properties of intestinal epithelium and can progress to infect the gut (Haraga et al., 2022).

A meta-genomic analysis by 16S rRNA sequencing and enterotype-based gut microbiota analysis showed that *Bacteroides*, *Proteobacteria*, and *Firmicutes* are the dominant bacterial phyla in healthy controls, as well as in patients with adenomatous polyps and CRC, with minor variations between groups. The relative abundance of *Fusobacterium* genus was significantly greater in CRC patients relative to normal subjects and to those with adenomas (Yang et al., 2019) as well as in tumour samples and stools from CRC patients (Bi et al., 2022).

**Figure 1.**
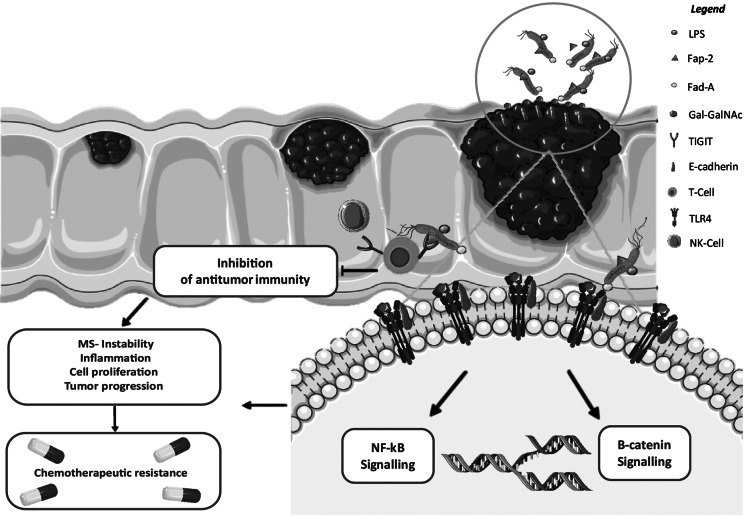
The entry of *Fn* into mucosal epithelial cells relies on surface molecules such as lipopolysaccharides (LPS), adhesin A (FadA), and fusobacterium autotransporter protein 2 (Fap2). *Fn* targets cells expressing Gal-Gal-Nac via FadA, binds to E-cadherin, and is internalized by epithelial cells. Once inside, *Fn* releases its RNA into the host cell cytoplasm, which is detected by cytosolic retinoic acid-inducible gene 1 (RIG-1), activating the β-catenin and NF-kB signalling pathways through FadA-E-cadherin binding on TLR4. This FadA-E-cadherin interaction accelerates carcinogenesis in the presence of predisposing mutations. Meanwhile, Fap2-TIGIT binding promotes tumour survival by inhibiting anti-tumour immunity and contributing to chemotherapeutic resistance. Figure created by https://smart.servier.com. Last accessed online on 18 July 2024.

The infiltration of *Fn* into mucosal epithelial cells would depend upon an impairment of the intestinal barrier, which allows *Fn* to adhere and invade gut cells. Surface molecules expressed by *Fn* include lipopolysaccharides (LPS), adhesin A (FadA), and fusobacterium autotransporter protein 2 (Fap2), which approaches cells expressing Gal-GalNAc and binds to E-cadherin, leading to internalization within epithelial cells. *Fn* is also capable of releasing RNA into the host cell cytoplasm, where it is subsequently detected by cytosolic sensor retinoic acid-inducible gene 1 (*RIG-1*). By triggering the activation of β-catenin and NF-kB signalling pathways (Zhou et al., 2018). Through FadA-E-cadherin binding on Toll-like receptor 4 (TLR4), *Fn* can accelerate colonic carcinogenesis mainly in the presence of pre-existing genetic alterations, while Fap2-TIGIT binding can promote tumour survival by smouldering anti-tumour immunity (Gur et al., 2015; Borroni et al., 2019) (Figure 1).

However, a recent paper by Zepeda-Rivera et al. sheds new light on the colonization capability of the CRC niche by *Fn* subspecies, confirming considerable strain-to-strain genotypic and phenotypic variations and revealing that a selected clade shows patho-adaptation to CRC. Such group, within *Fn* subspecies *animalis* (*Fna*) was referred to as clade C2 as opposed to C1, the latter representing the main oral pathobiont. Their work moved from pangenomic analysis of *Fusobacterium* strains obtained from targeted culture from human CRC specimens compared to strains from the oral cavity of non-cancerous subjects. First, at the subspecies level, it emerged that *Fna* is significantly enriched in CRC specimens, whereas the *nucleatum* subspecies is predominantly found in the oral niche. Virulence factors such as *fp1A*, a phospholipase autotransporter binding to host phosphoinosite-signalling lipids, and *fadA* are not significantly associated with *Fna* in comparison to other subspecies. Within *Fna* subspecies, the average nucleotide identity between the clades C1 and C2 ranged from 91.6% to 93.1% (95% being the threshold for species identity). While C1 and C2 have similar conserved core genomes, their accessory genomes vary, C2 having a larger one. The differences for C2 included larger chromosomes, more plasmids, and mobile genetic elements. The clades were also epigenetically distinct, with divergent methylome profiles. While the C2 was the only clade enriched in CRC, the representation of both clades did not differ in the oral cavity of CRC patients. While some virulence factors, such as *fadA* and *fpIA*, did not differ between the clades, other adhesins did, with *fap2*, *cmpA*, and *fusolisin* being enriched in *Fna* C2. Morphologically, *FnaC2* cells appear more fusiform and showed a higher level of invasion in HCT116 CRC cell line (noticeably a typical MSI cell-line). Furthermore, C2 possesses enhanced scavenger mechanisms and metabolic potential, which also contribute to patho-adaptation to the intestine and CRC niche. Eventually, in the dextran sodium sulfate-induced colitis *ApcMin^+/−^* mouse model of colorectal tumourigenesis, the exposure to (orally introduced) *FnaC2* caused a significant increase in adenomas compared to exposure to *FnaC1.* In confirmatory sequencing analysis of CRC specimens, only *Fna*C2 was significantly enriched therein (Zepeda-Rivera et al., 2024).

A relevant issue concerns the timing and involvement of *Fn* infection along the development and progression of CRC. It remains to be established whether the infection acts as an initiating event of colorectal tumourigenesis, rather than like a sort of “second-hit” event, accelerating tumour progression (Nakatsu et al., 2015; Rubinstein et al., 2019).

## Association between *Fn* and colorectal cancer according to pathological and molecular features

### CRC molecular subtypes: microsatellite and methylation status

To assess whether *Fn* is enriched in specific molecular subtypes of CRC, Tahara *et al.* assessed its association with MS and methylation status, as well with mutations in *BRAF*, *KRAS*, *TP53*, *CHD7*, and *CHD8* genes (Tahara et al., 2014). Most CRCs (74%) harboured the bacterial genome, which was heavily enriched in 5.4% of the cases. This subset of tumours was more likely to show MSI, with *MLH1* hypermethylation, *TP53* wild-type, and mutations in *CHD7* or *CHD8.* The shared CIMP^+^ molecular profile of *Fn* CRCs supported its pathogenic role in this subset of tumours.

A clear association of *Fn* with MSI-high, *MLH1* methylation, and the CIMP-pathway in CRCs also emerged in the work by Ito *et al*. They reported a higher *Fn* content in CRCs (56%) than in pre-malignant lesions (ranging from 30% to 35% in sessile serrated adenomas), in which the bacterium was more frequently detected in CIMP-high lesions than in CIMP-low/zero lesions (Ito et al., 2015). *Fn* positivity increased gradually from sigmoid colon to cecum in Sessile Serrated Adenomas (SSAs), but not in CRC. In a later and larger study, the proportion of *Fn*-high CRCs increased from rectal (2.5%; 4/157) to cecal cancers (11%; 19/178), with a significant linear trend along all subsites, independent of the association with MSI-high and CIMP^+^ tumours (Tahara et al., 2014). An independent study addressing the abundance of *Fn* in SSAs confirmed that up to 80% of proximal SSAs harbour invasive *Fn*, which is three times the frequency encountered in tubular adenomas, irrespectively of their location. While the prevalence of *Fn* in biofilms might be similar in different colon districts, the prevalence of invasive *Fn* in right-sided cancers approached 90% versus 42% of distal ones, and no relationship was found between biofilm positivity and tumour invasion by *Fn.* Interestingly, these authors also reported the presence of *Fn* in all tested metastatic lymph nodes (Yu et al., 2016). Data concerning the *Fn* abundance and CRC molecular subtypes are reported in Table 2.

**Table 2.** *Fn* abundance and CRC molecular features

	CRC type/mutation variables	*Fn* expression						
Study		Negative	(%)	Low	(%)	High	(%)	P-value
Tahara et al. (2014) (n = 149)	Cases	135			(90.6)	14	(9.4)	
	*KRAS* mutation							
	Wild-type	77			(51.7)	10	(6.7)	–
	Mutant	57			(38.3)	4	(2.7)	0.40
	*BRAF* mutation							
	Wild-type	103			(69.1)	8	(5.4)	–
	Mutant	27			(18.1)	6	(4.0)	0.09
	*PIK3CA* mutation							
	Wild-type	NA				NA		NA
	Mutant	NA				NA		NA
	*MLH1* methylation							
	Unmethylated	103			(69.1)	5	(3.4)	***–***
	Methylated	32			(21.5)	9	(6.0)	*0.0028*
	CIMP status							
	CIMP-low/neg	102			(68.5)	5	(3.4)	*0.001*
	CIMP-high	33			(22.1)	9	(6.0)	*–*
	MSI							
	MSS/MSI-low	68			(45.6)	3	(2.0)	***–***
	MSI-high	34			(28.8)	8	(5.4)	*0.018*
Ito et al. (2015) (n = 511)	Cases	225	(44)	143	(28)	143	(28)	
	*KRAS* mutation							
	Wild-type	157	(30.7)	101	(19.8)	98	(19.2)	0.93
	Mutant	68	(13.3)	42	(8.2)	45	(8.8)	
	*BRAF* mutation							
	Wild-type	216	(42.3)	136	(26.6)	131	(25.6)	0.20
	Mutant	9	(1.8)	7	(1.4)	12	(2.3)	
	*PIK3CA* mutation							
	Wild-type	205	(40.1)	126	(24.7)	119	(23.3)	0.079
	Mutant	20	(3.9)	16	(3.1)	24	(4.7)	
	*MLH1* methylation							
	Unmethylated	186	(36.4)	114	(22.3)	103	(20.2)	*0.0079*
	Methylated	22	(4.3)	19	(3.7)	31	(6.0)	
	CIMP status							
	CIMP-low/neg	203	(39.7)	127	(24.9)	113	(22.1)	*0.0088*
	CIMP-high	13	(2.5)	14	(2.7)	22	(4.3)	
	MSI							
	MSS/MSI-low	215	(42.1)	132	(25.8)	123	(24.1)	*0.0056*
	MSI-high	10	(2.0)	11	(2.2)	20	(3.9)	
Mima et al. (2015) (n = 598)	Cases	522	(87.4)	38	(6.3)	38	(6.3)	
	*KRAS* mutation							0.48
	Wild-type	307	(51.3)	18	(3.0)	21	(3.5)	
	Mutant	209	(34.9)	18	(3.0)	17	(2.8)	
	*BRAF mutation*							0.08
	Wild-type	439	(73.4)	31	(5.2)	27	(4.5)	
	Mutant	76	(12.7)	5	(0.8)	11	(1.8)	
	*PIK3CA* mutation							0.83
	Wild-type	404	(67.6)	25	(4.2)	30	(5.0)	
	Mutant	75	(12.5)	6	(1.0)	5	(0.8)	
	*MLH1* methylation							*<0.0001*
	Unmethylated	463	(77.4)	30	(5.0)	24	(4.0)	
	Methylated	53	(8.9)	7	(1.2)	14	(2.3)	
	CIMP status							*0.0009*
	CIMP-low/neg	441	(73.8)	30	(5.0)	23	(3.8)	
	CIMP-high	75	(12.5)	7	(1.2)	15	(2.5)	
	MSI							*<0.0001*
	MSS/MSI-low	447	(74.8)	24	(4.0)	22	(3.6)	
	MSI-high	66	(11.0)	12	(2.0)	16	(2.7)	
Mima et al. (2016) (n=1069)	Cases	935	(87.4)	67	(6.3)	67	(6.3)	
	*KRAS* mutation							0.44
	Wild-type	499	(46.7)	29	(2.7)	38	(3.5)	
	Mutant	370	(34.6)	29	(2.7)	24	(2.2)	
	*BRAF mutation*							*0.0009*
	Wild-type	771	(72.1)	50	(4.7)	45	(4.2)	
	Mutant	130	(12.2)	14	(1.3)	21	(2.0)	
	*PIK3CA* mutation							0.88
	Wild-type	713	(66.7)	48	(4.5)	52	(4.9)	
	Mutant	136	(12.7)	10	(0.9)	11	(1.0)	
	*MLH1* methylation							*<0.0001*
	Unmethylated	759	(71.0)	48	(4.5)	37	(3.5)	
	Methylated	96	(9.0)	13	(1.2)	25	(2.3)	
	CIMP status							*<0.0001*
	CIMP-low/neg	716	(70.0)	48	(4.5)	36	(3.4)	
	CIMP-high	139	(13.0)	13	(1.2)	26	(2.4)	
	MSI							*<0.0001*
	MSS/MSI-low	780	(73.0)	42	(3.9)	26	(2.4)	
	MSI-high	114	(10.7)	22	(2.1)	29	(2.7)	
L. Liu et al. (2018) (n = 951)	Cases	836	(87.9)	115			(12.1)	
	*KRAS* mutation							
	Wild-type	NA		NA				NA
	Mutant	NA		NA				
	*BRAF* mutation							
	Wild-type	NA		NA				NA
	Mutant	NA		NA				
	*PIK3CA* mutation							
	Wild-type	NA		NA				NA
	Mutant	NA		NA				
	*MLH1* methylation							
	Unmethylated	NA		NA				NA
	Methylated	NA		NA				
	CIMP status							
	CIMP-low/neg	NA		NA				NA
	CIMP-high	NA		NA				
	MSI							
	MSS/MSI-low	699	(73.5)	68			(7.1)	NS
	MSI-high	100	(10.5)	43			(4.5)	
Y. Chen et al. (2019) (n = 91)	Cases	66			(72.5)	25	(27.5)	
	*KRAS* mutation							
	Wild-type	30			(33.0)	11	(12.1)	
	Mutant	36			(39.5)	14	(15.4)	
	*BRAF* mutation							
	Wild-type	60			(65.9)	24	(26.4)	
	Mutant	6			(6.6)	1	(1.1)	
	*PIK3CA* mutation							
	Wild-type	NA				NA		
	Mutant	NA				NA		
	*MLH1* methylation							
	Unmethylated	NA				NA		
	Methylated	NA				NA		
	CIMP status							
	CIMP-low/neg	NA				NA		
	CIMP-high	NA				NA		
	MSI							
	MSS/MSI-low	26			(28.6)	4	(4.4)	
	MSI-high	40			(43.9)	21	(23.1)	
de Carvalho et al. (2019) (n = 152)	Cases	117	(77.0)	17	(11.2)	18	(11.8)	
	*KRAS* mutation							
	Wild-type	NA		NA		NA		
	Mutant	NA		NA		NA		
	*BRAF* mutation^a^							*<0.0001*
	Wild-type	113	(74.3)	13	(8.6)	14^a^	(9.2)	
	Mutant	3	(2.0)	3	(2.0)	5^a^	(3.3)	
	*PIK3CA* mutation							
	Wild-type	NA		NA		NA		
	Mutant	NA		NA		NA		
	*MLH1* methylation							*<0.0001*
	Unmethylated	101	(66.5)	8	(5.3)	8	(5.3)	
	Methylated	5	(3.3)	5	(3.3)	7	(4.6)	
	CIMP status							
	CIMP-low/neg	NA		NA		NA		
	CIMP-high	NA		NA		NA		
	MSI							*<0.0001*
	MSS/MSI-low	110	(72.4)	11	(7.2)	10	(6.5)	
	MSI-high	7	(4.6)	6	(4.0)	8	(5.3)	
Haruki et al. (2020) (n = 724)	Cases	625	(86.3)	50	(6.9)	49	(7.8)	
	*KRAS* mutation							0.16
	Wild-type	363	(50.1)	21	(2.9)	30	(4.1)	
	Mutant	246	(34.0)	25	(3.5)	18	(2.5)	
	*BRAF* mutation							*0.009*
	Wild-type	523	(72.2)	40	(5.5)	34	(4.7)	
	Mutant	86	(11.9)	8	(1.1)	15	(2.1)	
	*PIK3CA* mutation							0.66
	Wild-type	493	(68.1)	36	(5.0)	37	(5.1)	
	Mutant	85	(11.7)	7	(1.0)	9	(1.2)	
	*MLH1* methylation							NA
	Unmethylated	NA	NA	NA	NA	NA	NA	
	Methylated	NA	NA	NA	NA	NA	NA	
	CIMP status							*<0.001*
	CIMP-low/neg	484	(68.9)	35	(4.8)	27	(3.7)	
	CIMP-high	89	(12.3)	10	(1.4)	19	(2.6)	
	MSI							*<0.001*
	MSS/MSI-low	527	(72.8)	32	(4.4)	29	(4.0)	
	MSI-high	80	(11.0)	16	(2.2)	20	(2.8)	
Joo et al. (2024) (n = 1696^b^)	Cases	1440	(84.9)	151		(8.9)		
	*KRAS* mutation							0.29
	Wild-type	933	(55.0)	102		(6.0)		
	Mutant	401	(23.6)	35		(2.1)		
	*BRAF* mutation							*0.0008*
	Wild-type	1225	(72.2)	113		(6.7)		
	Mutant	162	(9.6)	31		(1.8)		
	*PIK3CA* mutation							NA
	Wild-type	NA	NA	NA		NA		
	Mutant	NA	NA	NA		NA		
	*MLH1* methylation							
	Unmethylated	NA	NA	NA		NA		
	Methylated	76	(5.3)	26		(17.2)		*<0.0001*
	CIMP status							*<0.0001*
	CIMP-low/neg	1198	(70.6)	112		(6.6)		
	CIMP-high	131	(7.7)	31		(1.8)		
	MSI							
	MSS/MSI-low	NA	NA	NA		NA		
	MSI-high	164	(11.4)	52		(34.4)		*<0.0001*

*Note:* The percentages were calculated in relation to the number of subjects who tested negative/low and high *Fn* out of the total number of subjects evaluated for each individual study. There are “missed” data with respect to the total negative/low and high *Fn*. All p-value<0.05 reported in italic format are statistically significant

Abbreviations: NA= Not Available; NS= Not Statistically significant.

a The total number of High positive cases exceeds the value reported in the summary.

b 106/1696 samples with unknown intratumoural presences and tumour characteristics treated as NA and excluded from the statistical testing.

Interestingly, employing NGS to explore the microbiome composition of CRC and its precursor lesions, Liu *et al.* showed that microbial communities, comprising *Fusobacterium* (and other CRC-associated pathobions, alike *Bacteroides*, *Parvimonas*, and *Prevotella*) are heterogeneous within a single neoplasia and change along the adenoma-carcinoma progression. Remarkably, taxa significantly enriched in CRC are spread along the whole tumour, although microbial communities do not differ from adjacent normal mucosa. The fold difference in the coefficient of variation for *Fusobacterium* was similar in adenomas and CRCs, indicating that its abundance is not affected during the transition from adenoma to cancer. However, the authors found differences in the microbiome composition between CRC with and without *KRAS* mutations, as well as between MSI and MSS ones. In the latter distinction, however, *Fn* was not among the species enriched in MSI CRCs (Liu et al., 2021). By a similar approach, a recent study employing NGS found elevated levels of *Fn* in CRC classified as CMS1 (which are typically MSI-high), but also in samples classifiable as CMS3 which are metabolic driven. In cell co-cultures, *Fn* was found to be pro-tumourigenic and pro-invasive, and it altered the metabolic profile. Thus, *Fn* can speed up the progression of CMS3 CRC, which is enriched in metabolic pathways involving cholesterol and proteoglycan metabolism and is characterized by an upregulation of inflammatory-related signalling pathways, such as IL-8 signalling or Th17 activation (Ternes et al., 2022). Additionally, *Fn* enhanced glutamine metabolism and formate secretion, the latter increasing cancer invasiveness and stemness.

Haruki et al. (2020) explored whether the expression of markers of autophagy by CRC cells could be associated with tumour content of *Fn.* The study assessed the expression of *BECN1* (beclin 1), *MAP1LC3* (LC3), and *SQSTM1* (p62) in tumour cells and their association with the amount of *Fn*, adjusted for potential molecular confounders (i.e. MSI, CIMP, and *BRAF* mutations). CRCs with intermediate/high expressed *BECN1* are characterized by low-load of *Fn*, suggesting a possible role for the autophagy in the elimination of this strain (Table 3). However, none of the autophagy proteins was associated with overall survival or with CRC-specific survival.

**Table 3.** Data by Haruki et al. (2020)

	Amount of *F. nucleatum* DNA in colorectal cancer tissue							Ordinal logistic regression model by amount of *F. nucleatum* DNA in colorectal cancer tissue			
	Negative (n = 625)	(%)	Low (n = 50)	(%)	High (n = 49)	(%)		Univariate OR	(95% CI)	Multivariable OR	(95% CI)
Tumour BECN1 expression (n = 628)											
Low	77	(12.3)	14	(28.0)	11	(22.4)		1	(Ref)	1	(Ref)
Intermediate	251	(40.2)	20	(0.40)	18	(36.7)		0.48	(0.26–0.88)	0.54	(0.29–0.99)
High	216	(34.5)	11	(22.0)	10	(20.4)		0.27	(0.14–0.52)	0.31	(0.16–0.60)
*P-value*	*<0.0001*						P-trend^a^	*<0.001*		*<0.001*	
Tumour MAP1LC3 expression (n = 689)											
Low	163	(26.1)	17	(34.0)	21	(42.9)		1	(Ref)	1	(Ref)
Intermediate	199	(31.8)	12	(24.0)	10	(20.4)		0.46	(0.25–0.83)	0.46	(0.25–0.85)
High	234	(37.4)	18	(36.0)	15	(30.6)		0.55	(0.32–0.95)	0.57	(0.33–0.99)
*P-value*	0.062						P-trend^a^	*0.043*		0.061	
Tumour SQSTM1 expression (n = 674)											
Low	131	(21.0)	15	(30.0)	12	(24.5)		1	(Ref)	1	(Ref)
Intermediate	278	(44.5)	18	(36.0)	13	(26.5)		0.54	(0.30–0.97)	0.55	(0.30–1.01)
High	176	(28.2)	13	(26.0)	18	(36.7)		0.88	(0.48–1.60)	0.89	(0.48–1.65)
*P-value*	0.83						P-trend^a^	0.75		0.79	

a P-trend was calculated by the linear trend across the ordinal categories of tumour BECN1, MAP1LC3, and SQSTM1 immunohistochemical expression level (low, intermediate, and high, as an ordinal predictor variable) in the IPW-adjusted ordinal logistic regression model for the amount of *Fn* DNA (negative, low, and high, as an ordinal outcome variable). All p<0.05 reported in italic format are statistically significant

The mentioned studies establish that approximately 1 out of 5 CRCs harbours a high copy number of *Fn*, has been found to cluster in MSI-high cancers, particularly those that are CIMP^+^ with *BRAF* mutations. With respect to MSI-high CRC, it would be advisable to stratify the *Fn* load using a state-of-the-art classification, comprising Lynch syndrome, Lynch-like cases, and sporadic ones. *Fn* was initially considered a potential environmental factor in the development of elevated MSI CRCs (Liu et al., 2018).

Most recently, the work by Joo *et al.* clarified that both hereditary and sporadic MMR-deficient CRCs are prone to *Fn* colonization. They confirmed the significant correlation between *Fn* and *BRAF* p.V600E somatic mutation and CIMP-high CRC (Table 2), yet the *Fn* enrichment in hereditary cancers also indicates the importance of the MMR-deficient tumour microenvironment for the colonization, irrespectively of the origin of the defect (Joo et al., 2024).

Accordingly, the main molecular feature of CRC associated with *Fn* colonization is MSI. One of the key reasons for investigating this relationship is the differential immune responses observed in MSI-H and MSS tumours. MSI-H tumours are known for their strong immune activation, characterized by high levels of tumour-infiltrating lymphocytes (TILs), due to their high mutational burden, which generates numerous neoantigens.

### Relationship between Fn and immune response in the tumour microenvironment

The role of *Fn* in shaping the immune response within the CRC tumour microenvironment has been extensively studied, particularly in relation to T-cell infiltration and macrophage modulation.

The relationship between *Fn* abundance and the density of infiltrating T-cells in CRC was assessed in a large series of pathological specimens from Nurses’ Health Study and the Health Professionals Follow-up Study. In the study by Mima et al. (2016), 13% (134 out of 1069) of CRCs showed high levels of *Fn* DNA, more frequently in MSI-high (18%) tumours than in MSI-low or MSS ones (4% of cases). They confirmed the over-representations of *MLH1* hypermethylation and CIMP-high status among *Fn*-high CRCs, together with their right-sidedness, while the association with female sex and *BRAF* mutation did not reach statistical significance. As to T-cell populations, the amount of *Fn* was inversely associated with CD3^+^T-cell density in CRC tissues, while no differences were reported for CRC mortality (Mima et al., 2015) (Table 4).

**Table 4.** Data by Mima et al. (2015)

	All patients (n = 598)		The amount of *Fn* in colorectal carcinoma tissue						
T-Cells density by quartile		(%)	Negative (n = 522)	(%)	Low (n = 38)	(%)	High (n = 38)	(%)	P-value
CD3^+^									*0.012*
Q.le 1 (0–85 cells/mm^2^)	142	(23.8)	117	(22.4)	9	(23.7)	16	(42.1)	
Q.le 2 (86–210 cells/mm^2^)	141	(23.6)	122	(23.4)	13	(34.2)	6	(15.8)	
Q.le 3 (211–425 cells/mm^2^)	142	(23.8)	126	(24.1)	7	(18.4)	9	(23.7)	
Q.le 4 (≥426 cells/mm^2^)	142	(23.8)	130	(24.9)	6	(15.8)	6	(15.8)	
CD8^+^									0.11
Q.le 1 (0–46 cells/mm^2^)	140	(23.4)	117	(22.4)	12	(31.6)	11	(29)	
Q.le 2 (47–131 cells/mm^2^)	140	(23.4)	118	(22.6)	11	(29)	11	(29)	
Q.le 3 (132–320 cells/mm^2^)	140	(23.4)	129	(24.7)	4	(10.5)	7	(18.4)	
Q.le 4 (≥320 cells/mm^2^)	140	(23.4)	124	(23.6)	8	(21)	8	(21)	
CD45R0^+^									0.56
Q.le 1 (0–184 cells/mm^2^)	144	(24.1)	124	(23.6)	11	(29)	9	(23.7)	
Q.le 2 (185–427 cells/mm^2^)	143	(23.9)	128	(24.5)	5	(13.2)	10	(26.3)	
Q.le 3 (428–895 cells/mm^2^)	143	(23.9)	130	(24.9)	6	(15.8)	7	(18.4)	
Q.le 4 (≥896 cells/mm^2^)	144	(24.1)	119	(22.8)	14	(36.8)	11	(29)	
FOXP3^+^									*0.038*
Q.le 1 (0–10 cells/mm^2^)	137	(22.9)	117	(22.4)	7	(18.4)	13	(34.2)	
Q.le 2 (11–23 cells/mm^2^)	137	(22.9)	118	(22.6)	6	(15.8)	13	(34.2)	
Q.le 3 (24–48 cells/mm^2^)	137	(22.9)	117	(22.4)	11	(29)	9	(23.7)	
Q.le 4 (≥49 cells/mm^2^)	137	(22.9)	126	(24.1)	9	(23.7)	2	(5.3)	

All p-value<0.05 reported in italic format are statistically significant

Lee et al. (2021) reported that, among MSI CRCs, the *Fn*-high subset was composed of larger tumours with deeper pT stage (as to the prevalence of pT3–pT4 tumours), which harboured less dense FoxP3^+^ TILs (at tumour centre and invasive margin) but denser CD163^+^ tumour associated macrophages in the tumour centre (Cavalleri et al., 2019). These findings reveal a different composition of the immune infiltrate (with pro-tumour steering of macrophages) associated with *Fn* colonization of MSI CRCs.

Due to the well-established association of low densities of CD3^+^T-cells and poor CRC prognosis (Grizzi et al., 2013; Laghi et al., 2020), Mima et al. (2015) later expanded the sample size to assess the association between *Fn*, T-cell infiltration, and patient outcomes. The study, comprising over 1000 CRCs, confirmed that the amount of *Fn* was significantly associated only with MSI-high molecular subtype at multivariable statistical analysis, independently of CIMP and *BRAF* CRC status, which were significant only at univariable statistical analyses. The survival of patients with *Fn*-high CRC was shorter than that of patients with *Fn^-^* cancers, even when stratified by stage. This finding, although in agreement with the low infiltration by T cells, was counterintuitive with respect to the better prognosis of patients with MSI CRC, usually ascribed to their lower metastatic potential (Malesci et al., 2007; Laghi & Malesci, 2012).

Consistent with previous findings, Hamada *et al.* tested whether the association between *Fn* and the magnitude of the adaptive immune response differs according to CRC MS-status. Accordingly, histopathologic lymphocytic reactions (i.e. the densities of CD3^+^, CD8^+^, CD45RO^+^, and FOXP3^+^ lymphocytes) were assessed in CRC strata differentiated by MS-status (Hamada et al., 2018) (Table 5). Surprisingly, high loads of *Fn* were negatively associated with TIL amounts in MSI-high tumours (multivariable OR, 0.45; 95% CI 0.22–0.92), but positively associated with the same variable in MSS tumours (significant interaction, adjusted by molecular confounders). In any case, patients with high loads of *Fn* in their tumours had an increased risk of disease-specific mortality, irrespective of MS-status (HR=1.27 for non-MSI-high CRCs, HR=2.23 for MSI-high CRCs at multivariable analysis) (Hamada et al., 2018) (Table 6). Currently, it remains unanswered how the bacterium can worsen the survival in the context of tumour groups differing for the type of genetic instability and the amount of immune response. One possible interpretation of this behaviour might lie in a smouldering activity of *Fn* towards adaptive immune response in MSI-high CRCs with abundant neo-antigens, as opposed to its pro-inflammatory properties in MSS cancers.

**Table 5.** Data by Hamada et al. (2018)

		Univariable OR	(95% CI)	Multivariable OR	(95% CI)
Model for CD3^+^ cell density (*n* = 550)					
Non-MSI-high	*Fn* ^-^	1	(Ref)	1	(Ref)
	*Fn* ^+^	0.82	(0.44–1.54)	0.76	(0.40–1.44)
MSI-high	*Fn* ^-^	1	(Ref)	1	(Ref)
	*Fn* ^+^	0.43	(0.17–1.10)	0.41	(0.16–1.05)
*P*_interaction_		0.27		0.28	
Model for CD8^+^ cell density (*n* = 543)					
Non-MSI-high	*Fn* ^-^	1	(Ref)	1	(Ref)
	*Fn* ^+^	0.81	(0.43–1.53)	0.86	(0.45–1.63)
MSI-high	*Fn* ^-^	1	(Ref)	1	(Ref)
	*Fn* ^+^	0.38	(0.15–0.97)	0.42	(0.16–1.08)
*P*_interaction_		0.19		0.22	
Model for CD45RO^+^ cell density (*n* = 558)					
Non-MSI-high	*Fn* ^-^	1	(Ref)	1	(Ref)
	*Fn* ^+^	1.01	(0.54–1.90)	1.05	(0.56–1.97)
MSI-high	*Fn* ^-^	1	(Ref)	1	(Ref)
	*Fn* ^+^	0.85	(0.32–2.21)	0.87	(0.33–2.28)
*P*_interaction_		0.76		0.75	
Model for FOXP3^+^ cell density (*n* = 530)					
Non-MSI-high	*Fn* ^-^	1	(Ref)	1	(Ref)
	*Fn* ^+^	0.65	(0.34–1.25)	0.62	(0.32–1.21)
MSI-high	*Fn* ^-^	1	(Ref)	1	(Ref)
	*Fn* ^+^	1.65	(0.64–4.29)	1.52	(0.58–3.98)
*P*_interaction_		0.11		0.14

**Table 6.** Data by Hamada et al. (2018)

		CRC-specific mortality					Overall mortality				
	Tot. no.	No. events	UnivariateHR	(95% CI)	Multivariate HR	(95% CI)	No.events	UnivariateHR	(95% CI)	MultivariateHR	(95% CI)
Non-MSI-high											
*Fn* ^-^	785	242	1	(Ref)	1	(Ref)	456	1	(Ref)	1	(Ref)
*Fn* ^+^	76	34	1.57	(1.09–2.24)	1.27	(0.89–1.83)	45	1.20	(0.89–1.63)	0.99	(0.73–1.35)
MSI-high											
*Fn* ^-^	118	12	1	(Ref)	1	(Ref)	63	1	(Ref)	1	(Ref)
*Fn* ^+^	58	13	2.50	(1.14–5.47)	2.23	(1.01–4.89)	32	1.26	(0.82–1.93)	1.30	(0.85–2.00)
*P*_interaction_^b^			0.29		0.20			0.86		0.31	

However, increasing evidence suggests that other immune cell types, such as natural killer (NK) cells, neutrophils, and dendritic cells (DCs), are also influenced by the presence of *Fn* in CRC tissues.

NK cells are critical for anti-tumour immunity through their cytotoxic activity and cytokine production. However, *Fn* has been implicated in immune evasion strategies that impair NK cell function. Studies suggest that *Fn* may downregulate NK cell activation markers or promote the expression of immune checkpoint molecules, such as TIGIT, which inhibits NK cell-mediated cytotoxicity (Gur et al., 2015). Additionally, the presence of *Fn*-derived lipopolysaccharides (LPS) can induce an immunosuppressive cytokine profile, reducing NK cell activation and favouring tumour immune escape (Borroni et al., 2019).

Recent research indicates that *Fn* can drive the activation of neutrophil extracellular traps (NETs), web-like structures composed of DNA and antimicrobial proteins that neutrophils release in response to infection or tumour signals (Kong et al., 2023). In CRC, NETs have been shown to promote EMT, facilitating tumour invasion and metastasis (Salvucci et al., 2022). Furthermore, *Fn* infection leads to increased recruitment of tumour-associated neutrophils (TANs), which are often associated with poor prognosis in CRC patients (Yan et al., 2017).

DCs are key antigen-presenting cells that initiate and regulate adaptive immune responses. However, *Fn* has been shown to impair DC function, reducing their ability to stimulate anti-tumour T-cell responses (Borroni et al., 2019). In CRC, *Fn*-colonized tumours exhibit reduced infiltration of mature DCs and an accumulation of tolerogenic DC subsets, which suppress effective immune responses (Gholizadeh et al., 2017). This may be mediated by *Fn*-induced activation of the β-catenin and NF-κB pathways, which favour immune suppression over activation (Zhou et al., 2018).

Understanding the role of *Fn* in modulating NK cells, neutrophils, and dendritic cells provides new insights into its contribution to immune evasion and tumour progression in CRC. Future research should focus on therapeutic strategies targeting these immune interactions, such as modulating neutrophil activation, restoring NK cell cytotoxicity, or enhancing DC antigen presentation. These findings support the broader role of *Fn* in shaping the immune landscape of CRC beyond T-cell infiltration, offering potential biomarkers and therapeutic targets for improving CRC treatment outcomes.

## Environmental factors: diet, cancer, and *Fn* load

The interplay between environmental factors and CRC risk extends beyond genetic predisposition, with increasing evidence highlighting the influence of diet and microbiome alterations. Among these factors, diet has received particular attention due to its direct role in modulating gut microbiota composition, including *Fn* colonization levels. Epidemiological studies suggest that diets rich in fibre support beneficial gut bacteria and may reduce *Fn* load, thereby lowering CRC risk (Mehta et al., 2017). Conversely, pro-inflammatory diets (e.g. high consumption of processed and red meats) have been associated with an increased abundance of *Fn*, heightened inflammatory responses, and a greater likelihood of *Fn*-positive CRC (Liu et al., 2018).

Beyond diet, other environmental factors may also contribute to *Fn* enrichment in the gut microbiome. Studies indicate that smoking, alcohol consumption, and antibiotic use can significantly alter gut bacterial diversity and create an environment favouring pathogenic bacteria like *Fn* (Gholizadeh et al., 2017). Additionally, urbanization and industrial pollutants may impact gut homeostasis, potentially increasing susceptibility to *Fn*-associated tumourigenesis (Alexander et al., 2018). Recent research has also proposed a role for *Fn*-derived bacterial metabolites, such as riboflavin, in shaping the tumour microenvironment by activating mucosal-associated invariant T (MAIT) cells, which drive pro-inflammatory signalling linked to CRC progression (Li et al., 2020).

Besides the link between high *Fn* loads and CRC molecular profiles, extrinsic factors contributing to CRC such as diet composition may also be associated with tumour bacterial content. Indirect evidence for such an association came from two studies. The first one showed that adherence to a prudent diet (enriched in whole grains and dietary fibre) rather than to a Western-type diet was associated with a reduced risk of developing *Fn* ^+^ cancers, irrespectively of tumour location, in U.S. prospective cohorts (Mehta et al., 2017). The authors suggested that the cancer preventive effect of a diet enriched in dietary fibres may be modulated by the microbiota, as assessed through the load of *Fn* (Table 7). The second study evaluated the association between the intake of foods sustaining the release of inflammatory cytokines (IL-6 and TNF receptor superfamily member 1B) and high levels of C-reactive protein with the risk of developing CRC stratified by *Fn* content (Liu et al., 2018). Inflammatory effects of the diet were estimated by empirical dietary inflammatory pattern score, a system in which high scores correspond to an inflammatory diet and high plasma levels of IL-6, TNF receptor superfamily member 1B, and C-reactive protein. Higher scores were associated with an increased risk of *Fn* ^+^ CRC, again in the proximal colon, supporting an interaction between an inflammatory diet and the microbiota. As a component of the gut ecosystem, *Fn* residency could be influenced by alimentary habits in driving colonic carcinogenesis (Liu et al., 2018) (Table 8).

**Table 7.** Data by Mehta et al. (2017)

		Tertiles of EDIP^a^ scores					
		Quartile 1	Quartile 2	Quartile 3	Quartile 4	P-trend^b^	P-Heterogeneity^c^
Tumour *Fn* status		Prudent dietary pattern					
High (n = 60)		20	10	20	10		
	*Multivariable* *HR (95% CI)* ^d^	1 (Ref)	0.47 (0.22–1.00)	0.92 (0.49–1.73)	0.43 (0.20–0.92)	0.08	
Low (n = 65)		23	16	14	12		*0.03*
	*Multivariable* *HR (95% CI)* ^d^	1 (Ref)	0.65 (0.34–1.23)	0.52 (0.26–1.01)	0.43 (0.22–0.87)	*0.02*	
Negative (n = 894)		207	222	234	231		
	*Multivariable* *HR (95% CI)* ^d^	1 (Ref)	1.04 (0.86–1.26)	1.00 (0.83–1.22)	0.95 (0.77–1.17)	0.47	
		Western dietary pattern					
High (n = 60)		13	18	17	12		
	*Multivariable* *HR (95% CI)* ^d^	1 (Ref)	1.44 (0.70–2.95)	1.49 (0.70–3.09)	1.16 (0.52–2.60)	0.72	
							0.19
Low (n = 65)		12	15	16	22	*0.02*	
	*Multivariable* *HR (95% CI)* ^d^	1 (Ref)	1.29 (0.60–2.78)	1.50 (0.70–3.20)	2.25 (1.08–4.66)		
Negative (n = 894)		219	242	210	223	0.12	
	*Multivariable* *HR (95% CI)* ^d^	1 (Ref)	1.20 (0.99–1.44)	1.08 (0.88–1.33)	1.25 (0.99–1.58)		

All p-value<0.05 reported in italic format are statistically significant. Abbreviations: CI, Confidence Interval; HR, Hazard Ratio.

a Empirical dietary inflammatory pattern (EDIP) scores.

b Tests for trend were conducted using the median value of each quartile category as a continuous variable.

c We tested for heterogeneity by using a likelihood ratio test, comparing a model that allows separate associations for the two CRC.

d Stratified by age, calendar years, and gender and adjusted for total caloric intake (kcal/day), family history of colorectal cancer in any first-degree relative, history of previous endoscopy, pack-years of smoking (never, 0–4, 5–19, 20–39, or >40), body mass index (kg/m2), physical activity (MET-hours/week), and regular aspirin or NSAID use (≥2 tablets/week).

**Table 8.** Data by Liu et al. (2018)

			Tertiles of EDIP^a^ scores				
			T1 (lowest)	T2	T3 (highest)	P_trend_	P_Heterogeneity_
CRC site	Tumour *Fn* status						
Proximal (n = 463)	Negative (n = 396)		136	138	122		*0.003*
		*Age-adjusted HR (95% CI)*	1 (Ref)	1.00 (0.79–1.27)	0.94 (0.74–1.21)	0.72	
		*Multivariable HR (95% CI)*	1 (Ref)	1.01(0.79–1.29)	0.96 (0.74–1.24)	0.84	
	Positive (n = 67)		13	24	30		
		*Age-adjusted HR (95% CI)*	1 (Ref)	1.92 (0.97–3.79)	2.59 (1.35–4.98)	*0.003*	
		*Multivariable* *HR (95% CI)*	1 (Ref)	1.94(0.98–3.84)	2.61 (1.35–5.05)	*0.003*	
Distal (n = 272)	Negative (n = 253)		76	88	89		0.35
		*Age-adjusted HR (95% CI)*	1 (Ref)	1.15 (0.85–1.57)	1.24 (0.91–1.69)	0.21	
		*Multivariable HR (95% CI)*	1 (Ref)	1.25 (0.91–1.72)	1.32 (0.95–1.83)	0.13	
	Positive (n = 19)		8	6	5		
		*Age-adjusted HR (95% CI)*	1 (Ref)	0.72 (0.25–2.10)	0.68 (0.22–2.08)	0.47	
		*Multivariable HR (95% CI)*	1 (Ref)	0.81 (0.27–2.37)	0.76 (0.25–2.34)	0.60	
Rectal (n = 202)	Negative (n = 178)		65	58	55		0.49
		*Age-adjusted HR (95% CI)*	1 (Ref)	0.91 (0.64–1.31)	0.92 (0.64–1.32)	0.65	
		*Multivariable(95% CI)*	1 (Ref)	0.95 (0.66–1.37)	0.94 (0.64–1.39)	0.79	
	Positive (n = 24)		9	5	10		
		*Age-adjusted HR (95% CI)*	1 (Ref)	0.53 (0.18–1.59)	1.25 (0.51–3.11)	0.59	
		*Multivariable HR (95% CI)*	1 (Ref)	0.56 (0.19–1.69)	1.31 (0.52–3.27)	0.54	
MSI status							
Non-MSI-high (n = 999)	Negative (n = 699)		225	247	227		
		*Age-adjusted HR (95% CI)*	1 (Ref)	1.10 (0.92–1.31)	1.07 (0.89–1.29)	0.47	
		*Multivariable HR (95% CI)*	1 (Ref)	1.15 (0.95–1.38)	1.12 (0.92–1.36)	0.25	
	Positive (n = 68)		22	19	27		
		*Age-adjusted HR (95% CI)*	1 (Ref)	0.84 (0.45–1.56)	1.37 (0.77–2.41)	0.26	
		*Multivariable HR (95% CI)*	1 (Ref)	0.88 (0.47–1.63)	1.44 (0.82–2.55)	0.19	
MSI-high (n = 187)	Negative (n = 100)		35	36	29		
		*Age-adjusted HR (95% CI)*	1 (Ref)	1.00 (0.63–1.60)	0.87 (0.53–1.42)	0.65	
		*Multivariable HR (95% CI)*	1 (Ref)	1.04 (0.65–1.67)	0.90 (0.54–1.48)	0.76	
	Positive (n = 43)		9	18	16		
		*Age-adjusted HR (95% CI)*	1 (Ref)	2.01 (0.90–4.49)	1.95 (0.86–4.43)	0.11	
		*Multivariable HR (95% CI)*	1 (Ref)	2.10 (0.94–4.70)	2.01 (0.88–4.56)	0.09	

All p-value<0.05 reported in italic format are statistically significant. Abbreviations: CI, Confidence Interval; HR, Hazard Ratio.

a Empirical dietary inflammatory pattern (EDIP) scores.

The association between various dietary factors and faecal *Fn* was evaluated in a cross-sectional Japanese study involving healthy adults without a history of colorectal cancer or precancerous lesions. A high intake of dairy products in healthy adults may reduce *Fn* and prevent colorectal cancer (Narii et al., 2023). Although within its limitations, the study by Shimomura *et al.* found that the abundance of *Fn* had a significant natural indirect effect on CRC risk based on their highest fibre intake compared to the lowest fibre intake (Shimomura et al., 2023).

Recently, a new model of CRC pathogenesis was developed involving the bacterial riboflavin synthase from the enriched colorectal bacteria, i.e. *Fn.* Its role in CRC development may be attributed to microbe-derived riboflavin metabolites activating mucosal-associated invariant T-Cell (MAIT) (Li et al., 2020), which produce proinflammatory cytokines and cytotoxic molecules integral to the pathological process of CRC (Wang et al., 2022).

Sikavi *et al.* hypothesized that the association between a diet linked to a greater abundance of sulfur-metabolizing bacteria and distal CRC is moved toward specific molecular subtypes and/or relative enrichment (or depletion) of intra-tumoural CRC-associated microbes. Their data did not provide conclusive evidence to support their hypothesis, and they were unable to draw significant conclusions about the relationship between dietary content and molecular tumour status in CRC. The only emerging difference was the negative association with *Bifidobacterium spp.* of distal CRC in men (Sikavi et al., 2021).

While this section primarily examines dietary influences, it is crucial to recognize that a combination of environmental exposures likely contributes to *Fn* colonization, gut dysbiosis, and CRC development. Future research should investigate how various environmental factors interact with dietary patterns to shape *Fn*-mediated tumourigenesis. A deeper understanding of these relationships could guide preventive strategies, including dietary modifications and microbiome-targeted interventions, to mitigate the risk of *Fn*-associated CRC.

## *Fn* and outcome of CRC patients

### Pathological features of *Fn* ^*+*^ tumours and their role as prognostic biomarkers

When considering the association with patient demographics, no significant associations emerged as to the relationship of *Fn* amount with patient age or sex. Besides the above-mentioned association with right-sided MSI CRC, in a model adjusted for patient age, sex, CRC location, and BMI, patients with high faecal *Fn* abundance had a 3-fold increased likelihood of being diagnosed with rectal compared with colon tumours, and 5-fold increased risk compared with right-sided CRC (Eisele et al., 2021).

As to tumour pathological features, the rate of *Fn* ^+^ usually increases with the depth of tumour invasion (T), poor tumour differentiation (de Carvalho et al., 2019; Haruki et al., 2020; Joo et al., 2024; Liu et al., 2018; Mima et al., 2015; Sun et al., 2016; Wei et al., 2016), neural invasion, and with the presence of nodal (N) (Castellarin et al., 2012; Yamaoka et al., 2018; Yan et al., 2017), and distant metastasis (M) (Chen et al., 2022), although such results have not been entirely confirmed (Tahara et al., 2014; Ito et al., 2015; Chen et al., 2019, 2022; Eisele et al., 2021). Contrarily, it is worth noting that Nakatsu *et al.*, investigating the microbial communities at different stages of carcinogenesis, found that *Fn* was predominantly enriched in the early stage (i.e. stage I–II) of CRC (Nakatsu et al., 2015). Data related to *Fn* abundances, demographics, and pathological features in CRC are reported in Table 9.

**Table 9.** *Fn* abundance, demographics and pathological features in CRC

		The amount of *Fn* DNA in colorectal cancer tissue						
Study	Socio-demographic variables and CRC clinical outcome	Negative	(%)^a^	Low	(%)^a^	High	(%)^a^	*P-value*
Tahara et al. (2014) (n = 149)	Cases	135		(90.6)		14	(9.4)	
	Age (years)							
	≤70	81		(54.3)		5	(3.4)	-
	>70	53		(35.6)		9	(6.0)	0.09
	Gender							
	Men	82		(55.0)		7	(4.7)	-
	Women	53		(33.6)		7	(4.7)	0.57
	Tumour location							
	Proximal colon	63		(42.3)		9	(6.0)	0.20
	Distal colon	47		(31.5)		2	(1.3)	-
	AJCC disease stage							
	I–II	NA		NA		NA	NA	
	III–IV	NA		NA		NA	NA	
	pT stage (depth of tumour invasion)							NA
	pT1 (submucosa)	NA		NA		NA	NA	
	pT2 (muscularis propria)	NA		NA		NA	NA	
	pT3 (subserosa)	NA		NA		NA	NA	
	pT4 (serosa or other organs)	NA		NA		NA	NA	
	Tumour differentiation							NA
	Well to moderate	NA		NA		NA	NA	
	Poor	NA		NA		NA	NA	
Ito et al. (2015) (n = 511)	Cases	225	(44)	143	(28)	143	(28)	
	Mean age ± SD (years)	66.1 ± 11.9		69.1 ± 10.9		66.5 ± 12.2		0.088
	Gender							
	Men	127	(24.9)	82	(16.1)	77	(15.1)	0.82
	Women	98	(19.1)	61	(11.9)	66	(12.9)	
	Tumour location							
	Proximal colon	64	(12.5)	54	(10.6)	52	(10.2)	0.11
	Distal colon	161	(31.5)	89	(17.4)	90	(17.6)	
	AJCC disease stage							
	I–II	86	(16.8)	64	(12.5)	66	(12.9)	0.23
	III–IV	139	(27.2)	79	(15.5)	77	(15.1)	
	pT stage (depth of tumour invasion)							NA
	pT1 (submucosa)	NA	NA	NA	NA	NA	NA	
	pT2 (muscularis propria)	NA	NA	NA	NA	NA	NA	
	pT3 (subserosa)	NA	NA	NA	NA	NA	NA	
	pT4 (serosa or other organs)	NA	NA	NA	NA	NA	NA	
	Tumour differentiation							NA
	Well to moderate	NA	NA	NA	NA	NA	NA	
	Poor	NA	NA	NA	NA	NA	NA	
Mima et al. (2015) (n = 598)	Cases	522	(87.3)	38	(6.4)	38	(6.3)	
	Mean age ± SD (years)	67.2 ± 8.5		67.7 ± 7.4		66.8 ± 7.3		0.89
	Gender							0.08
	Men	184	(30.8)	11	(1.9)	7	(1.1)	
	Women	338	(56.5)	27	(4.5)	31	(5.2)	
	Tumour location							*0.006*
	Caecum	81	(13.6)	7	(1.2)	15	(2.5)	
	Ascending to transverse colon	172	(28.8)	12	(2.0)	13	(2.1)	
	Splenic flexure to sigmoid	154	(25.8)	7	(1.2)	7	(1.2)	
	Rectosigmoid and rectum	113	(18.8)	12	(2.0)	3	(0.5)	
	AJCC disease stage							*0.003*
	I–II	279	(46.7)	14	(2.3)	21	(3.5)	
	III-IV	213	(35.6)	23	(3.8)	15	(2.5)	
	pT stage (depth of tumour invasion)							NA
	pT1 (submucosa)	NA	NA	NA	NA	NA	NA	
	pT2 (muscularis propria)	NA	NA	NA	NA	NA	NA	
	pT3 (subserosa)	NA	NA	NA	NA	NA	NA	
	pT4 (serosa or other organs)	NA	NA	NA	NA	NA	NA	
	Tumour differentiation							*0.002*
	Well to moderate	484	(80.9)	30	(5.0)	30	(5.0)	
	Poor	38	(6.4)	7	(1.2)	8	(1.3)	
Mima et al. (2016) (n = 1,069)	Cases	935	(87.4)	67	(6.3)	67	(6.3)	
	Mean age ± SD (years)	69.2 ± 8.8		71.1 ± 9.0		68.8 ± 8.3		0.21
	Gender							0.36
	Men	400	(37.4)	26	(2.5)	23	(2.2)	
	Women	535	(50.0)	41	(3.8)	44	(4.1)	
	Tumour location							*0.001*
	Caecum	145	(13.6)	13	(1.2)	20	(1.9)	
	Ascending to transverse colon	296	(27.7)	23	(2.2)	27	(2.5)	
	Splenic flexure to sigmoid	287	(26.9)	12	(1.1)	12	(1.1)	
	Rectosigmoid and rectum	205	(19.2)	19	(1.8)	7	(0.7)	
	AJCC disease stage							*0.003*
	I–II	499	(46.7)	32	(3.0)	35	(3.3)	
	III–IV	354	(33.1)	30	(2.8)	27	(2.5)	
	pT stage (depth of tumour invasion)							*0.0008*
	pT1 (submucosa)	93	(8.7)	1	(0.009)	5	(0.5)	
	pT2 (muscularis propria)	189	(17.7)	12	(1.1)	4	(0.4)	
	pT3 (subserosa)	532	(49.7)	41	(3.8)	47	(4.4)	
	pT4 (serosa or other organs)	44	(4.1)	8	(0.8)	5	(0.5)	
	Tumour differentiation							*< 0.0001*
	Well to moderate	862	(80.6)	55	(5.1)	48	(4.5)	
	Poor	72	(6.7)	11	(1.0)	19	(1.8)	
Sun et al. (2016) (n = 152)	Cases	34	(22.4)	NA	NA	118	(77.6)	
	Age (years)							NS
	<60	12	(8.0)	NA	NA	57	(37.5)	
	≥60	22	(14.4)	NA	NA	61	(40.1)	
	Gender							NS
	Men	20	(13.2)	NA	NA	75	(49.3)	
	Women	14	(9.2)	NA	NA	43	(28.3)	
	Tumour location							NS
	Right-sided	7	(4.6)	NA	NA	42	(27.6)	
	Left-sided	13	(8.6)	NA	NA	40	(26.3)	
	Rectum	14	(9.2)	NA	NA	36	(23.7)	
	pT stage (depth of tumour invasion)							*<0.001*
	pT1 (submucosa)	4	(2.6)	NA	NA	2	(1.3)	
	pT2 (muscularis propria)	5	(3.3)	NA	NA	16	(10.5)	
	pT3 (subserosa)	13	(8.5)	NA	NA	43	(28.3)	
	pT4 (serosa or other organs)	12	(7.9)	NA	NA	57	(37.5)	
	AJCC disease stage							*0.034*
	I	2	(1.3)	NA	NA	4	(2.6)	
	II	16	(10.5)	NA	NA	14	(9.2)	
	III	11	(7.3)	NA	NA	53	(34.9)	
	IV	5	(3.3)	NA	NA	20	(13.1)	
	Tumour differentiation							*<0.001*
	Well	2	(5.9)	NA	NA	5	(3.3)	
	Moderate	30	(88.2)	NA	NA	89	(58.6)	
	Poor	0	(0.0)	NA	NA	23	(15.1)	
L. Liu et al. (2018) (n=951)	Cases							
	Mean age ± SD (years)	NA			NA	NA	NA	NA
	Gender							NA
	Men	NA			NA	NA	NA	
	Women	NA			NA	NA	NA	
	Tumour location							*0.01*
	Proximal colon	396			(41.6)	67	(7.0)	*0.003*
	Distal colon	431			(45.3)	43	(4.5)	
	AJCC disease stage							NA
	I–II	NA			NA	NA	NA	
	III–IV	NA			NA	NA	NA	
	pT stage (depth of tumour invasion)							NA
	pT1 (submucosa)	NA			NA	NA	NA	
	pT2 (muscularis propria)	NA			NA	NA	NA	
	pT3 (subserosa)	NA			NA	NA	NA	
	pT4 (serosa or other organs)	NA			NA	NA	NA	
	Tumour differentiation							NA
	Well to moderate	NA			NA	NA	NA	
	Poor	NA			NA	NA	NA	
Y. Chen et al. (2019) (n=91)	Cases	66			(72.5)	25	(27.5)	
	Mean age ± SD (years)	58.52±10.421			58.72±12.638			0.298
	Gender							0.364
	Men	38			(41.6)	17	(18.7)	
	Women	28			(30.8)	8	(8.8)	
	Tumour location							0.549
	Ascending colon	13			(14.3)	2	(2.2)	
	Transverse colon	12			(13.2)	3	(3.3)	
	Descending colon	16			(17.6)	9	(9.9)	
	Sigmoid colon	15			(16.5)	7	(7.7)	
	Rectum	10			(10.9)	4	(4.4)	
	AJCC disease stage							0.64
	II	36			(39.6)	15	(16.5)	
	III	30			(32.9)	10	(11.0)	
	pT stage (depth of tumour invasion)							0.066
	pT1 (submucosa)	NA			NA	NA	NA	
	pT2 (muscularis propria)	4			(4.4)	3	(3.3)	
	pT3 (subserosa)	24			(26.4)	3	(3.3)	
	pT4 (serosa or other organs)	38			(41.7)	19	(20.9)	
	Tumour differentiation							0.689
	High	7			(7.7)	2	(2.2)	
	Moderate	44			(48.3)	19	(20.9)	
	Poor	15			(16.5)	4	(4.4)	
de Carvalho et al. (2019) (n=152)	Cases	117	(77.0)	17	(11.2)	18	(11.8)	
	Mean age ± SD (years)	60.63±13.7						
	Gender							
	Men	64	(42.1)	10	(6.6)	7	(4.6)	0.406
	Women	53	(34.9)	7	(4.6)	11	(7.2)	
	Tumour location							
	Proximal colon	21	(13.8)	7	(4.6)	10	(6.6)	*0.001*
	Distal colon	96	(63.2)	10	(6.6)	8	(5.2)	
	AJCC disease stage							
	0–I	39	(25.7)	2	(1.3)	2	(1.3)	0.089
	II–III	74	(48.7)	13	(8.6)	15	(9.9)	
	IV	4	(2.6)	2	(1.3)	0	(0.0)	
	pT stage (depth of tumour invasion)							
	Tis/T1/T2	47	(30.9)	2	(1.3)	4	(2.6)	*0.035*
	T3/T4 (a b)	70	(46.1)	15	(9.9)	14	(9.2)	
	Tumour differentiation							
	Well to moderate	110	(72.4)	17	(11.2)	12	(7.9)	*0.011*
	Poor	7	(4.6)	0	(0.0)	4	(2.6)	
Haruki et al. (2020) (n=724)	Cases	625	(86.3)	50	(6.9)	49	(7.8)	
	Mean age (years) ± CI	69.3±8.9		70.5±8.7		68.0±8.5		0.38
	Gender							0.14
	Men	264	(36.5)	23	(3.2)	14	(1.9)	
	Women	361	(49.9)	27	(3.7)	35	(4.8)	
	Tumour location							*0.02*
	Right-sided	209	(28.9)	18	(2.5)	17	(2.3)	
	Left-sided	180	(24.9)	7	(0.9)	10	(1.4)	
	Rectum	132	(18.2)	14	(1.9)	6	(0.8)	
	Caecum	102	(14.0)	11	(1.5)	16	(16.7)	
	pT stage (depth of tumour invasion)							*0.003*
	pT1 (submucosa)	52	(7.2)	1	(0.1)	2	(0.3)	
	pT2 (muscularis propria)	124	(17.1)	9	(1.2)	2	(0.3)	
	pT3 (subserosa)	373	(51.5)	33	(4.6)	37	(5.1)	
	pT4 (serosa or other organs)	27	(3.7)	4	(0.6)	6	(0.8)	
	AJCC disease stage							*0.008*
	I	141	(19.5)	6	(0.8)	3	(0.4)	
	II	187	(25.8)	16	(0.2)	20	(2.7)	
	III	165	(22.8)	21	(2.9)	13	(1.8)	
	IV	83	(3.5)	3	(0.4)	10	(1.4)	
	Tumour differentiation							*<0.001*
	Well to moderate	581	(80.2)	42	(5.8)	37	(5.1)	
	Poor	44	(6.1)	7	(0.9)	12	(1.7)	
Eisele et al. (2021) (n.105)	Cases	83			(79.0)	22	(21.0)	
	Age (years)							0.24
	<65	45			(42.8)	15	(14.3)	
	≥65	38			(36.2)	7	(6.7)	
	Gender							0.56
	Men	51			(48.6)	12	(11.4)	
	Women	32			(30.5)	10	(9.5)	
	Tumour location							0.06
	Proximal colon	29			(27.6)	4	(3.8)	0.14
	Distal colon	30			(28.6)	7	(6.7)	
	Rectosigmoid junction and Rectum	24			(22.8)	11	(10.5)	
	AJCC disease stage							0.43
	I–II	56			(53.3)	11	(10.5)	
	III-IV	27			(25.7)	11	(10.5)	
	pT stage (depth of tumour invasion)							NA
	pT1 (submucosa)	NA			NA	NA	NA	
	pT2 (muscularis propria)	NA			NA	NA	NA	
	pT3 (subserosa)	NA			NA	NA	NA	
	pT4 (serosa or other organs)	NA			NA	NA	NA	
	Tumour differentiation							0.60
	Well	9			(8.6)	2	(1.9)	
	Moderate	61			(58.1)	14	(13.3)	
	Poor	12			(11.4)	5	(4.8)	
Xue et al. (2021) (n=513)	Cases	NA	NA	119	(23.2)	96	(18.7)	
	Age (years)							0.90
	<60	NA	NA	63	(12.3)	50	(9.8)	
	≥60	NA	NA	56	(10.9)	46	(9.0)	
	Gender							0.17
	Men	NA	NA	86	(16.8)	61	(11.9)	
	Women	NA	NA	33	(6.4)	35	(6.8)	
	Adenoma location							0.57
	Proximal colon	NA	NA	41	(8.0)	28	(5.5)	
	Distal colon	NA	NA	61	(11.9)	50	(9.7)	
	Proximal and distal	NA	NA	17	(3.3)	18	(3.5)	
	Adenoma size							0.52
	<1cm	NA	NA	77	(15.0)	58	(11.3)	
	≥1cm	NA	NA	42	(8.2)	38	(7.4)	
	Histologic type							0.99
	Tubular	NA	NA	98	(19.1)	79	(15.4)	
	Tubulovillous	NA	NA	21	(4.0)	17	(3.3)	
	Villous	NA	NA	0	(0.0)	0	(0.0)	
	Advanced adenomas							0.49
	No	NA	NA	122	(23.8)	52	(10.1)	
	Yes	NA	NA	93	(18.1)	44	(8.6)	
	High-grade dysplasia							0.19
	No	NA	NA	116	(22.6)	90	(17.5)	
	Yes	NA	NA	3	(0.6)	6	(1.2)	
W.-D. Chen et al. (2022) (n=141)	Cases	79		(56)		62	(44)	
	Age (years)							0.20
	<62	43		(54)		27	(44)	
	≥62	36		(45)		35	(56)	
	Gender							0.929
	Men	49		(62)		38	(61)	
	Women	30		(48)		24	(39)	
	Tumour location							0.090
	Proximal colon	32		(41)		34	(55)	
	Distal colon	47		(59)		28	(45)	
	AJCC disease stage							*ND*
	I–II	NA		NA		*ND*	*ND*	
	III–IV	NA		NA		*ND*	*ND*	
	pT stage (depth of tumour invasion)							0.786
	pT1–pT2	22		(28)		16	(26)	
	pT3–pT4	57		(72)		46	(74)	
	Tumour differentiation							0.422
	Well	8		(10)		3	(5)	
	Moderate	47		(59)		36	(58)	
	Poor	24		(30)		23	(37)	
	Tumour size							0.316
	<4 cm	23		(29)		23	(37)	
	≥4 cm	56		(71)		39	(63)	
	Neurological invasion							*0.006*
	No	76		(96)		51	(82)	
	Yes	3		(4)		11	(18)	
	Regional lymphnode metastasis							*0.002*
	No	53		(67)		25	(27)	
	Yes	26		(33)		37	(60)	
	Distant metastasis							*0.034*
	No	74		(94)		51	(82)	
	Yes	5		(6)		11	(18)	
Joo et al. (2024) (n=1696^b^)	Cases	1440	(84.9)	151		(8.9)		
	Mean age (years) ± CI	56.0 ± 25.1		55.8 ± 27.0				0.79
	Gender							0.26
	Men	720	(42.5)	82		(5.0)		
	Women	720	(42.5)	69		(4.1)		
	Tumour location							
	Proximal colon	457	(27.0)	85		(5.01)		
	Distal colon	406	(23.9)	31		(1.8)		*<0.0001*
	AJCC disease stage							
	I–II	NA	NA	NA		NA		NA
	III–IV	NA	NA	NA		NA		NA
	pT stage (depth of tumour invasion)							
	pT1–pT2	NA	NA	NA		NA		NA
	pT3–pT4	NA	NA	NA		NA		NA
	Tumour differentiation							*<0.0001*
	Well	270	(15.9)	51		(3.0)		
	Moderate							
	Poor	1127	(66.5)	97		(5.7)		
	Tumour size							
	<4 cm	NA	NA	NA		NA		NA
	≥4 cm	NA	NA	NA		NA		NA
	Neurological invasion							
	No	NA	NA	NA		NA		NA
	Yes	NA	NA	NA		NA		NA
	Regional lymphnode metastasis							
	No	NA	NA	NA		NA		NA
	Yes	NA	NA	NA		NA		NA
	Distant metastasis							
	No	NA	NA	NA		NA		NA
	Yes	NA	NA	NA		NA		NA

All p-value<0.05 reported in italic format are statistically significant. Abbreviations: NA= Not Available; NS= Not Statistically significant.

a The percentages were calculated in relation to the number of subjects who tested negative/low and high *Fn* out of the total number of subjects evaluated for each individual study. There are “missed” data with respect to the total negative/low and high *Fn.*

b 106/1696 samples with unknown intratumoural presences and tumour characteristics treated as NA and excluded from the statistical testing.

A study from China also reported a significant association with TNM components, including metastases (Sun et al., 2016).

Data from a small European cohort (Kunzmann et al., 2019) found an increased risk of death (overall survival; HR 1.68; 95% CI, 1.02–2.77; p=0.04) only after adjusting for age, TNM stage and adjuvant treatments; interestingly, the inclusion of MS-status in the model led to lose statistical significance (HR 1.80; 95% CI 0.97–3.28, p = 0.06). Differently, a North European (Bundgaard-Nielsen et al., 2019) study on a smaller cohort failed to find any significant association between *Fn* and CRC or adenoma (Table 10) and their outcome at 5 years. Eventually, in a study from South America, *Fn*-high content was also associated with a higher TNM stage (Table 11) and worse patient CRC-specific survival (de Carvalho et al., 2019).

**Table 10.** Data by Bundgaard-Nielsen et al. (2019)

*Fn* detection in normal tissue and different types of colonic lesions								
	CRC (tumours) (n = 99)	(%)	CRC (paired normal tissue) (n = 76)	(%)	Adenomas (n = 96)	(%)	Diverticula (n = 104)	(%)
*Fn* detection	29	(29.3)	22	(29.3)	3	(3)	33	(31.7)
Feature								
Mean age ± SD (years)	71 ± 10.1		70.5 ± 9.9		66 ± 11.7		63 ± 14.0	
Age groups								
<40	0	(0.0)	0	(0.0)	1	(1.0)	3	(2.9)
40–49	2	(2.0)	1	(1.3)	6	(6.3)	16	(15.4)
50–59	8	(8.1)	5	(6.6)	18	(18.8)	24	(23.1)
60–69	35	(35.4)	28	(36.8)	30	(31.3)	25	(24.0)
70–79	28	(28.3)	22	(28.9)	25	(26.0)	17	(16.3)
80–89	22	(22.2)	16	(21.1)	15	(15.6)	19	(18.3)
>90	4	(4.0)	4	(5.3)	1	(1.0)	0	(0.0)
Sex								
Men	44	(44.4)	41	(53.9)	47	(48.0)	55	(52.9)
Women	55	(55.6)	35	(46.1)	51	(52.0)	49	(47.1)
Tumour location								
Right colon	49	(49.5)	41	(53.9)	15	(15.6)	4	(3.8)
Left colon	48	(48.5)	34	(44.7)	73	(76.0)	93	(89.4)
Rectum	2	(2.0)	1	(1.3)	3	(3.1)	0	(0.0)
Not reported	0	(0.0)	0	(0.0)	5	(5.2)	7	(6.7)
Disease stage								
Tumour stage								
I	7	(7.1)	7	(9.2)				
II	41	(41.4)	29	(38.2)				
III	43	(43.4)	32	(42.1)				
IV	8	(8.1)	8	(10.5)				
Subtype								
*Villous*					1	(1.0)		
*Tubulovillous*					6	(6.3)		
*Tubular*					89	(92.7)		
Dysplasia								
*Mild*					11	(11.5)		
*Moderate*					55	(57.3)		
*Severe*					30	(31.3)		
Inflammation								
*Diverticulosis*							23	(22.1)
*Diverticulitis*							81	(77.9)
								

*Note: P-*value, Not significant between each neoplasia and paired normal tissue.

**Table 11.** Data by de Carvalho et al. (2019)

Univariate odds ratio (OR) models adjusted by logistic regression of the impact of the amount of *Fn* in CRC tissue and clinical, pathological, and molecular data.			
Variables	Univariable OR	(95% CI)	P-value
Proximal *vs* distal colon			
*Fn* ^-^	1	(Ref)	
*Fn*-low	3.20	(1.27–9.38)	*0.034*
*Fn* ^+^	5.71	(2.01–16.2)	*0.001*
T3/T4 *vs* Tis/T1/T2			
*Fn* ^-^	1	(Ref)	
*Fn*-low	50.4	(1.10–23.05)	*0.037*
*Fn* ^+^	2.35	(0.73–7.58)	0.152
Poor *vs* well to moderate			
*Fn* ^-^	1	(Ref)	
*Fn*-low	NE		NE
*Fn* ^+^	5.24	(1.34–20.5)	*0.017*
MSI-positive *vs* MSI-negative			
*Fn* ^-^	1	(Ref)	
*Fn*-low	8.57	(2.45–30.05)	*0.001*
*Fn* ^+^	12.57	(3.77–41.88)	*<0.0001*
Mutated *vs* wild type *BRAF*			
*Fn* ^-^	1	(Ref)	
*Fn*-low	8.07	(1.48–43.92)	*0.016*
*Fn* ^+^	14.49	(3.10–67.72)	*0.0001*
Negative *vs* positive *MLH1*			
*Fn* ^-^	1	(Ref)	
*Fn*-low	12.63	(3.01–52.94)	*0.001*
*Fn* ^+^	17.68	(4.56–68.50)	*<0.0001*
Negative *vs* positive *MSH2*			
*Fn* ^-^	1	(Ref)	
*Fn*-low	19.09	(1.60–2270.86)	0.02
*Fn* ^+^	NE		NE
Negative *vs* positive *PMS2*			
*Fn* ^-^	1	(Ref)	
*Fn*-low	11.33	(2.41–53.10)	*0.002*
*Fn* ^+^	22.31	(5.37–92–65)	*<0.0001*

Ordinal logistic regression analysis to assess associations of tumour stage and MSI status with the amount of *Fn* DNA in CRC tissue			
Variables	Odds ratio	(95% CI)	*P-value*
*Fn* ^-^ *vs Fn*-low *vs Fn* ^+^			
Tumour stage (T3/T4 *vs.* Tis/T1/T2)	2.81	(1.04–7.55)	*0.028*
MSI (MSI-positive *vs* MSI-negative)	8.64	(3.33–22.39)	*<0.0001*

All p-value<0.05 reported in italic format are statistically significant. Abbreviations: MSI, microsatellite instability; CI, Confidence Interval; OR, Odds ratio; NE, Not Evaluated.

In the largest study, high loads of *Fn* had a negative association with CRC-specific mortality in a stage-stratified Cox model, indicating that *Fn* would act as a negative prognostic factor across stages, independently of other biomarkers. The prognostic associations of *Fn* with CRC outcome have also been the matter of two recent meta-analyses, both supporting the association of *Fn* with both shorter survival and higher CRC stage (Colov et al., 2020; Gethings-Behncke et al., 2020).

Besides the match with more advanced stage at diagnosis supporting an accelerating role for *Fn* in tumour progression, an interesting study evaluating the features of patients with/out metachronous adenomas after polypectomy detected a higher abundance of *Fn* in patients with metachronous adenomas than in those without. Multivariable analysis showed that a high abundance of *Fn*, male gender, and age were associated with metachronous adenomas (Xue et al., 2021). Such translational results are intriguing as they also would support a role for *Fn* in tumour development.

Overall, studies point to the association of *Fn* with worse outcomes and advanced CRC stage, yet it remains to be firmly established whether such association is stage-independent. If the prognostic association with worse CRC outcomes holds true across stages, *Fn* may act as a disease modifier, accelerating tumour progression and worsening prognosis (Table 12).

**Table 12.** *Fn* actionability as modifier in disease progression

Author	Comparison	Cases (N^a^)	TNM correlation	Survival	*P*-value
Castellarin et al. (2012)	*Fn* high *vs Fn* low	99	Higher N stage: *p=0.0035*	OS: no difference (data not shown) DFS: NA DSS: NA	NA
Flanagan et al. (2014)	*Fn* high-moderate/low	32	*Not available for the 32 cases (Table 1)*	OS: decreased for *Fn* high-moderate DFS: NA DSS: NA	*0.008*
Mima et al. (2015)	*Fn* ^-^ *vs Fn* high *vs Fn* low	598	Association with stage II and IV: *p≤0.003*	OS: no difference (data not shown) DFS: NA DSS: NA	NA
Wei et al. (2016)	*Fn* high *vs Fn* low	180	Stage I–II vs III–IV: p=0.101 Higher T-stage: *p=0.015* Higher N-stage: *p=0.011* Higher M-stage: p=0.718	OS: decreased for *Fn* high DFS: decreased for *Fn* high DSS: NA	*0.003* *0.001*
Mima et al. (2016)	*Fn* high/*Fn* low *vs* no *Fn*	1069	Higher T: *p=0.0008* Higher N: p=0.84 Higher stage: *p=0.003*	OS: no difference DFS: NA DSS: decreased for *Fn* high	0.99 *0.003*
Sun et al. (2016)	*Fn* high *vs Fn* low	152	Higher TNM: *p=0.034*	OS: decreased for *Fn* high DFS: NA DSS: NA	*0.027*
Yan et al. (2017)	*Fn* high *vs Fn* low	280	Study conducted on stage III–IV CRC Higher T: *p=0.015* Higher N: *p=0.008* Higher M: *p=0.020*	OS: NA DFS IIIA: no difference DSS IIIA: no difference DFS IIIB-C: decreased for *Fn* high DSS IIIB-C: decreased for *Fn* high DFS IV: decreased for *Fn* high DSS IV: decreased for *Fn* high	0.371 0.247 *≤0.048* *≤0.038* *0.019* *0.042*
Yu et al. (2017)	*Fn* high *vs Fn* low	265^b^	Comparison between recurrent CRC and non-recurrent CRC TNM correlation and % adjuvant treatment, not assessable	DFS: decreased in *Fn* high^b^	*<0.001*
Bullman et al. (2017)	*Fn* high *vs Fn* low	77	Not assessable	No association with recurrence	0.3
Yamaoka et al. (2018)	*Fn* high *vs Fn* low	100	Higher p Stage: p=0.071	OS: decreased for *Fn* high DFS: NA DSS: decreased for *Fn* high (stage IV)	*0.026* *0.041*
Hamada et al. (2018)	*Fn* ^+^ *vs Fn* ^-^	1041	NA	OS: no difference for *Fn+* in MSI high OS: no difference for *Fn+* in non-MSI high DFS: NA DSS: decreased for *Fn+* in MSI high DSS: decreased for *Fn+* in non-MSI high	0.23 0.25 *0.029* *0.013*
Lee et al. (2018)	*Fn* high *vs Fn* low	246	Conducted on adjuvant (stage III or HRII) and palliative (stage IV) Higher M: *p<0.001*	OS: decreased for palliative *Fn+* DFS: no difference for adjuvant DSS: NA	*0.042* 0.84
Bundgaard-Nielsen et al. (2019)	*Fn* ^+^ *vs Fn* ^-^	99	No association with stage	OS: no difference DFS: no difference DSS: NA	>0.05 >0.05
Chen et al. (2019)	*Fn* ^-^ *vs Fn* high *vs Fn* low	91	NA (study conducted on stage III or high-risk stage II CRC)	OS: decreased for *Fn* high DFS: NA DSS: NA	*0.038*
Kunzmann et al. (2019)	*Fn* ^-^ *vs Fn* high *vs Fn* low	190	Association with stage: p=0.238	OS: decreased for *Fn* high DFS: NA DSS: NA	*0.04*
Oh et al. (2019)	*Fn* high *vs Fn* low/neg	593	Higher T: *p=0.005* Higher N: p=0.464	OS: NA DFS: no difference for stage II/III DFS: increased for *Fn* high in non-sigmoid adjuvant DFS: no difference for sigmoid-rectal adjuvant DSS: NA	0.671 *0.026* 0.199
Leung et al. (2019)	Relative abundance *Fn*, comparison with non-tumour mucosa	19	pT only reported	OS: <20mo decreased OS: 20–40mo no difference OS: >40mo no difference DFS: NA DSS: NA	*0.018* NA NA
de Carvalho et al. (2019)	*Fn* high *vs Fn* low	152	Higher T: *p=0.035*	OS: decreased for *Fn* high DSS: decreased for *Fn* high	*0.037* *0.028*
Serna et al. (2020)	*Fn* positivity after neo-adjuvant therapy for LARC§	85	Relapse-free survival (Responsiveness to neo-adjuvant therapy)	HR 7.5 (CI 3–19)	*0.034*
Yi et al. (2021)	*Fn* positivity in responders and non-responders to nCRT	105	Patients with LARC and healthy individuals	Probability of response in training and validation cohorts	*<0.001* *0.003*
Lee et al. (2021)	*Fn* high *vs Fn* low/neg	126	Higher pT3–pT4	No association with recurrence	NA

All p-value<0.05 reported in italic format are statistically significant. Abbreviations: LARC, locally advanced rectal cancer; NA, Not Available; OS, Overall Survival; DFS, Disease Free Survival; DSS, Disease Short Survival; HR, Hazard Ratio.

a Independent of MS-status at multivariate analysis.

b Cumulative data from cohorts 2 (n=92) and 3 (n=173); p-value at multivariate independent from TNM stage (II *vs* III).

### Fn as a potential driver of mechanisms of tumour invasion

Similarly, it remains unclear whether *Fn* can also trigger epithelial-to-mesenchymal transition (EMT) in CRC cells, favouring their spread. EMT comprises the loss of E-cadherin accompanied by the expression of mesenchymal drivers, alike *TWIST1* (Celesti et al., 2013) and enhanced invasiveness. Ma et al. (2018) demonstrated that *Fn* promotes the proliferation and invasion of normal colon epithelial cells (NCM460) by interacting with E-cadherin without affecting its expression. In their study on stage III and IV CRCs, Yan *et al.* reported that the expression of EMT-related markers (N-cadherin) was associated with *Fn* loads, being shifted towards mesenchymal features in *Fn*-high CRCs, altogether with the expression of markers of stemness (Yan et al., 2017). Most recently, *in vitro* experiments by Kong *et al.* demonstrated that *Fn*-induced NETs indirectly accelerated malignant tumour growth through angiopoiesis and facilitate tumour metastasis via EMT-related cell migration, matrix metalloproteinase (MMP)-mediated degradation of basement membrane proteins, and the subsequent trapping and dissemination of CRC cells (Kong et al., 2023). Salvucci *et al.* observed a dysregulation of MAPK signalling, inducing EMT at the protein level, when comparing Fusobacteriales-high and Fusobacteriales-low patients in the TCGA-COAD-READ cohort. Their bioinformatic analysis reported that patients with mesenchymal tumours and a high prevalence of *Fn* have worse prognosis in CRC (Salvucci et al., 2022).

Zhang et al. found that, mechanistically, *Fn* activated the TGF-β1/SMAD signalling pathway to promote EMT *via* the miR-122-5p/FUT8 axis, stimulating CRC cells to excrete exosome-wrapped miR-122-5p, and activating the FUT8/TGF-b1/Smads axis to promote metastasis (Zhang et al., 2023).

Recently, new findings revealed that *Fn* bacterial ferritins that protect DNA from oxidative stress (*Fn*- DNA hunger/stationary phase protective proteins [Dps]) is a novel multifunctional *Fn* virulence factor that lyses and disrupts erythrocytes, enhances intracellular survival of *Fn* in macrophages, and promotes the migration of CRC cells via the CCL2/CCL7-induced EMT and CRC metastasis. A high level of serum anti-*Fn*-Dps antibody was found to be prevalent in populations, and elevated anti-I-Dps antibody levels were observed in CRC patients (Wu et al., 2023).

The mechanistic link between *Fn* and the enhancement of the EMT process as a route accelerating CRC progression is intriguing. However, it should be reconciled with the association with MSI CRC, which is characterized by a better outcome and consistently by a molecular profile with poor EMT features (Celesti et al., 2013).

### *Fn* as a candidate predictive biomarker: impact on CRC chemotherapy. Resistance mechanisms and therapeutic implications

Currently, the behaviour of CRC largely depends upon administered treatments, either surgical or medical, the latter both in adjuvant and neo-adjuvant settings.

Studies have shown that *Fn* can promote chemo-resistance through multiple mechanisms, including the activation of TLR4/MYD88 signalling, leading to autophagy-mediated survival of cancer cells (Yu et al., 2017). This is particularly relevant in patients undergoing 5-fluorouracil (5-FU) or oxaliplatin-based chemotherapy, where high *Fn* loads have been correlated with higher recurrence rates and reduced treatment efficacy. More recently, La Course et al. found that 5-FU has potent antibacterial activity against *Fn* CRC tumour isolates, suggesting that this treatment could inhibit the growth of these bacteria within tumours. Their study highlights the potential dual benefits of 5-FU as both an effective cancer treatment and a potent antimicrobial agent within the tumour microenvironment (LaCourse et al., 2022). Further research is needed to unravel the impact of 5-FU on the microbiota within tumour lesions and its implications for cancer therapy.

Accordingly, data concerning the association between patient outcome and *Fn* should be evaluated considering clinical settings involving the administration of chemotherapy. In Korean patients with either high-risk stage II or stage III CRC receiving adjuvant therapy (either FOLFOX or CAPOX), the abundance of *Fn* did not differentiate survival. However, subgroup analyses showed that in patients with cancer proximal to the sigmoid colon (i.e. cecal, ascending, transverse, and descending colon), those with *Fn*-high CRC had a better disease-free survival (Oh et al., 2019). In this subset, *Fn* was an independent prognostic factor at multivariable analysis (HR, 0.42; 95% CI, 0.18 to 0.97; p = 0.043). This favourable effect on patient outcome was restricted to non-MSI-high cancers. In another Korean study including patients who received chemotherapy (sample size, 246 patients), either with adjuvant or palliative intent, *Fn*-high was associated with poor overall survival in the palliative cohort (p = 0.042), also at multivariable analysis (adjusted HR 1.69 [95% CI 1.04–2.75], p = 0.034), while its amount was not associated with recurrence in the adjuvant cohort (Lee et al., 2018). One study from China (Chen et al., 2019), including high-risk stage II and stage III patients, reported an association between high *Fn* levels and worse outcomes in CRC patients (Table 3). The distribution of *Fn* ^+^ CRC was widespread along the colon, with a slightly more frequent in the descending segments. A similar worsening behaviour associated with abundance of *Fn* on the overall survival in patients with stage IV CRC was also reported in a small Japanese study (Yamaoka et al., 2018). Yan *et al.*, by studying stage III and IV CRCs, found that high loads of *Fn* correlated with local and nodal invasion, as well with distant metastasis, and discriminated a worse CRC-specific survival, irrespectively of TNM staging (Yan et al., 2017). Interestingly, patients with low *Fn* levels had a better disease-free survival than those with high *Fn* levels, if treated with adjuvant therapy (with significant interaction).

In their innovative paper, Yu *et al.* detected abundant *Fn* in CRCs of patients with recurrence after chemotherapy as compared to patients without. The load of *Fn* above a cut-off identified by ROC-curve analysis could predict recurrence (Yu et al., 2017), yet their analyses were adjusted for stage but not for adjuvant therapy. Noticeably, in cellular and xenograft models, *Fn* led to the development of chemo-resistance. Mechanistically, *Fn* enhanced the expression of *TLR4* and *MYD88* transcripts, inducing a selective loss of miR-18a* and miR-4802 expression, ultimately activating autophagy (Yu et al., 2017). Meanwhile, Bullmann *et al.* showed in another fascinating paper that *Fn* is maintained in distant metastases, documenting the stability of the microbiome between primary and secondary tumour lesions. As viable bacteria identical to those isolated from the primary cancer were retrieved and cultured from metastatic lesions, the persistence of *Fusobacterium* species remains clonal through the establishment of metastases. In an *in vivo* model, *Fn* persisted in patient-derived xenografts through multiple passages, and such bacteria were invasive once incubated with CRC cells. Eventually, treatment of the xenografts with metronidazole reduced the *Fusobacterium* load, cell proliferation, and tumour growth. However, the authors did not confirm the association between *Fusobacterium* load and CRC recurrence (Bullman et al., 2017).

Moreover, emerging evidence highlights the role of *Fn*-derived exosomal miRNAs (e.g. miR-1246, miR-92b-3p, and miR-27a-3p) in promoting EMT and metastasis. Guo et al. recently found that exosomes (tiny particles released by cells) from CRC cells infected with *Fn* help cancer spread by carrying specific miRNAs. When CRC cells (HCT116, MSI; SW480, MSS) were exposed to these exosomes, they moved more easily and changed shape, like an EMT process, which helps cancer spread. The miRNA content in these exosomes was different, with high levels of miR-1246, miR-92b-3p, and miR-27a-3p. These miRNAs increased cell movement by lowering GSK3b levels and reducing E-Cadherin while raising Vimentin levels. The exosomes also increased CXCL16 levels, further promoting migration. In both lab and animal models, exposure to these exosomes led to more tumour growth and liver metastases. In CRC patients, higher levels of exosomal CXCL16 and miR-1246/92b-3p/27a-3p were linked to more *Fn* and advanced cancer stages (Guo et al., 2021). These miRNAs may serve as potential biomarkers for identifying patients at higher risk of therapy resistance, suggesting a need for microbiome-based stratification in CRC treatment plans.

In summary, *Fn* plays a dual role in CRC chemotherapy responses both as a driver of resistance and as a potentially targetable component of treatment. Future studies should explore how modulating *Fn* levels through antibiotics, microbiome interventions, or targeted therapies could enhance chemotherapy efficacy and improve patient outcomes.

### Locally advanced rectal cancers treated with neo-adjuvant therapy

A translational paper investigated by RNA *in situ* hybridization (RNA-ISH), digital image analysis, and qPCR, the abundance of *Fn* in tumours from patients with locally advanced rectal cancer (LARC) treated with neoadjuvant chemotherapy (Serna et al., 2020), having a cohort of untreated patients as control. The concordance between the two approaches (qPCR and RNA-ISH) was high (agreement rate, 86%). *Fn* was mainly located at the luminal surface of the cancers, and its density was significantly higher in untreated cancer samples than in tumour specimens collected after treatment, with a positivity rate of 57% and 25%, respectively. Although *Fn* abundance did predict the responsiveness to treatment, the difference between the rates of responsiveness in *Fn* ^+^ (34%) and negative (13%) tumours only approached significance p=0.08. However, considering *Fn* status in specimens collected after treatment, 59% of patients with *Fn* ^+^ tumours relapsed, as compared to only 11% among *Fn^-^* ones (OR 11.6, 95% CI 3.2–43.3; p<0.001). Noteworthy, *Fn* drop after therapy was associated with better outcomes. Data on CD3^+^ and CD8^+^ TILs were also available for a subset of patients. While no difference was reported in TIL densities related to *Fn* status before surgery, CD8^+^ cells appeared to be higher in *Fn-*negative patients before and after treatment, suggesting that *Fn* persistence after neo-adjuvant therapy for LARC is associated with its relapse, possibly related to reduced immune cytotoxicity (Serna et al., 2020).

Shortly following the first report on the relevance of *Fn* as to the outcome of LARC treated with neoadjuvant therapy, another study explored the microbiome as a predictor of responsiveness to neoadjuvant therapy in LARC patients. In their prospective longitudinal study, Yi *et al.* also report several associations of the microbiome profile with LARC as well as with its responsiveness to neoadjuvant therapy. These almost parallel findings are rather surprising in light of the previously strengthened associations with right-sided, MSI colon cancers. In LARC, the composition of the microbiome investigated by NGS was different in patients and controls, mainly for the contribution of the pathobions in a subset of patients, referred to as having type 1 LARC. These patients also showed a significantly lower fraction of responders to neoadjuvant therapy than patients with type 2 LARC. Although *Fn* was significantly reduced post-therapy, the network of microbiome composition between pre- and post-therapy was associated with responsiveness, butyrate-producing bacteria being associated with a better response than that observed in patients colonized by *Coriobacteriaceae*, *Granulicatella*, *R. pickettii*, and *E. tayi* other than *Fusobacterium.* Accordingly, the authors developed a receiver operating characteristic (ROC) curve analysis for microbiome composition to predict therapy responsiveness, with valuable performances in both training and validation settings. Although numbers were small with respect to the number of analyzed variables, this work supports a new biological variable, possibly estimating the outcome of the patients with LARC treated with neoadjuvant therapy (Yi et al., 2021).

### Potential exploitation of *Fn* for the diagnosis of CRC

There are high expectations on the increasing exploitation of biomarkers, detectable in faeces or blood, that might help to improve the screening for CRC (Eklöf et al., 2017; Xie et al., 2017; Chung et al., 2024; Imperiale et al., 2024; Komaroff, 2024). Several studies have reported the potential value of *Fn* in implementing CRC diagnosis, suggesting that it might act as a diagnostic biomarker. Wong *et al.* reported that the quantification (by real-time PCR) of faecal *Fn* combined with a faecal immunochemical test (FIT) could improve the diagnosis of advanced adenoma and CRC. Specifically, *Fn* increased the sensitivity of FIT for the detection of both CRC (from 73% to 92%) and advanced adenoma (from 15.5% to 38.6%). In terms of copy number, the relative abundance of faecal *Fn* DNA in CRC and advanced adenoma groups was 132 and 3.8 folds higher than in the control group, respectively (Wong et al., 2017) (Table 13). Similarly, Liang *et al.* found that with faecal *Fn* alone, the sensitivity and specificity of CRC diagnosis was 82.0% and 80.7%, respectively, while the sensitivity increased to 92.8% when faecal *Fn* was used in combination with FIT plus testing for the DNA of other faecal bacteria (i.e. *Bacteroides clarus*, *Roseburia intestinalis*, *Clostridium hathewayi*, and one undefined species, labelled as *m7*) (Liang et al., 2017) (Table 13).

**Table 13.** Data by Liang et al. (2017), Wong et al. (2017)

	FIT			FIT^+^ *Fn*			
	Positive No.	Sensitivity (%)	Specificity (%); 95% CI	Positive No.	Sensitivity (%)	Specificity (%); 95% CI	*P*-value
Wong et al. (2017) – (Subject n = 309)							
Colorectal cancer (n = 104)	76	73.1 (64.4–81.8)	98.0 (95.1–100)	96	92.3 (86.5–97.1)	93.0 (88.0–97.0)	*<0.001*
* Stage I (n = 22)*	15	68.2 (50.0–86.4)		19	86.4 (72.7–100.0)		
* Stage II (n = 31)*	23	74.2 (58.1–90.3)		29	93.6 (83.9–100.0)		
* Stage III (n = 39)*	30	76.9 (64.1–89.7)		37	94.9 (87.2–100.0)		
* Stage IV (n = 12)*	8	66.7 (41.7–91.7)		11	91.7 (75.0–100.0)		
* Proximal (n = 28)*	23	82.1 (67.9–96.4)		26	92.9 (82.1–100.0)		
* Distal (n = 76)*	53	69.7 (59.2–80.3)		70	92.1 (85.5–97.4)		
Advanced adenoma (n = 103)	16	15.5 (8.7–22.3)		39	38.6 (28.7–48.5)		*0.007*
Q. Liang et al. (2017) – (Subject n = 230)							
Colorectal cancer (n = 111)	78	70.3	98.3	103	92.8	79.8^b^	*<0.001*
* Stage I (n = 17)*	7	41.2		13	76.5^a^		
* Stage II (n = 42)*	33	78.6		42	100.0		
* Stage III (n = 43)*	30	69.8		40	93.0		
* Stage IV (n = 9)*	8	88.9		8	88.9		

All p-value<0.05 reported in italic format are statistically significant. Abbreviations: CI, Confidence Interval.

a plus, other faecal bacteria (i.e. *Bacteroides clarus, Roseburia intestinalis, and Clostridium hathewayi*) Sensitivity = 82.4%.

b plus, other faecal bacteria (i.e. *Bacteroides clarus, Roseburia intestinalis, and Clostridium hathewayi*) Specificity = 81.5%.

Conversely, Aitchison *et al.* assessed the faecal amount of *Fn* in a cohort study involving 185 patients referred for FIT compared to CRC patients and age-matched controls (both n=57). The rate of positivity was higher in patients undergoing FIT and in CRC patients (47%) than in controls (7%; p<0.001), but no association was found between the carriage of *Fn* and FIT positivity (p=0.59). However, the presence of *Fn* in the stools was associated with an enhanced risk of finding colonic neoplastic lesions at colonoscopy (O.R. 3.1; p=0.02) (Aitchison et al., 2022).

It should be critically considered whether these promising results support the implementation of the molecular detection of *Fn* DNA in stool-based tests to be comparatively assessed with other molecular approaches improving the diagnostic yield of FIT (Imperiale et al., 2014, 2024; Komaroff, 2024).

However, published meta-analyses do not support unequivocal conclusions, as two would support *Fn* as a good diagnostic marker (Amitay et al., 2017; Yu et al., 2017; Peng et al., 2018), and another found data less encouraging (Huang et al., 2018), and a third one found comparative metrics (Sze & Schloss, 2018) discouraging.

Most recently, and although with limitations, Zhang *et al.* suggested salivary *Fn* DNA as a non-invasive potential biomarker for the detection and prognosis of CRC. They showed that the relative level of *Fn* DNA was increased in the saliva of CRC patients compared to subjects with clean colonoscopies, hyperplastic polyps, or adenomas. Besides, *Fn* DNA had a performance in CRC diagnosis superior to carcinoembryonic antigen and carbohydrate antigen 19-9, its levels also being associated with overall and disease-free survival of the patients (Zhang et al., 2022).

However, to properly address these issues requires very large numbers, avoiding population bias, and additional translational efforts are needed before embarking on such large studies.

### *Fn* as a potential molecular target for CRC treatment

In gastroenterology, phage therapy has been addressed mainly in infectious diseases, such as cholera. Currently, it is being explored in the eradication of *Fn* in CRC. Intestinal microbiota undergoes significant modifications in CRC (Kannen et al., 2019). Zheng *et al.* observed detrimental over-population of *Fn* in mice and patients, suppressing the beneficial butyrate-producing *Clostridium butyricum.* In human saliva, the authors isolated a temperate (i.e. lysogenic) phage capable of targeting and killing *Fn* without impacting the *Clostridium butyricum* population, thus reporting the occurrence of a natural system selectively controlling bacterial proliferation, which may turn into a possible therapeutic strategy. Additionally, they developed a phage-guided biotic–abiotic hybrid nano-system that could increase the chemotherapeutic potency of Irinotecan against CRC cells, whilst also selectively killing the *Fn* population, thus allowing at the same time the expansion of butyrate-producing bacteria (Zheng et al., 2019). Another report showed that *Fn* can modulate signalling pathways and activate autophagy, which may play a key role in mediating CRC chemoresistance. As mentioned, the authors demonstrated that *Fn* was significantly more represented in the CRC tissue of patients who had a post-chemotherapy recurrence of disease compared to those without recurrence. Such a contribution of *Fn* to chemoresistance can be mechanistically explained by interference with the TLR4 receptor, MYD88 signalling, and autophagy activation involving miRNAs. Among the latter, the reduction of miR-4802 and of miR-18a* is implicated in the accumulation of autophagosomes, indicating that *Fn* could activate autophagy through the miRNA modulation (Yu et al., 2017).

In HCT116 MSI CRC cells, *Fn* was able to stimulate proliferation and migration and up-regulated the expression of c-MET, which in turn, if knocked-down, reduced such stimulation. Either endogenous or exogenous miR-139–5p weakened the effects of *Fn* in this cancer cell system, similar to c-MET knockdown, indicating that miRNAs are involved in mediating *Fn*-induced effects (Zhao et al., 2020). These different modulations in the response to therapy suggest *Fn* as a potential molecular target.

Accordingly, ongoing clinical trials are investigating therapeutic strategies to target this bacterium. A Phase 2 clinical trial involves the use of oral metronidazole to reduce *Fn* levels in CRC tissues, with the goal of assessing its impact on tumour cells and the surrounding microenvironment (Oncology Institute of Southern Switzerland, 2024).

Another ongoing study explores the potential benefits of combining metronidazole with postoperative chemotherapy in patients with high levels of *Fn.* Researchers aim to determine whether reducing this bacterium can enhance the effectiveness of chemotherapy in CRC treatment (Fang, 2020).

These studies highlight the growing interest in targeting *Fn* as a potential strategy to improve colorectal cancer treatment outcomes.

## Concluding remarks

Among the components of the gut microbiome, *Fn* has gained attention for its association with colonic neoplasia and with given molecular subtypes, particularly MSI-high CRC. The parallel associations with *BRAF-*mutated and CIMP^+^ tumours suggested that it may be involved in the development of sporadic MMR defects. This hypothesis has been refuted by suggesting a possible contribution of the molecular pathway of both hereditary and sporadic MMRd CRC subtypes in *Fn* colonization, independently of its role.

*Fn* high loads have also been associated with advanced stages and worse prognosis, suggesting that *Fn* can act as a disease modifier, enhancing CRC progression and reducing patient survival. Several data point to its capability of smouldering anti-tumour immunity while eliciting pro-tumoural inflammatory responses, which would contribute to its involvement in tumour progression. However, it remains to be established whether it acts as a driver of carcinogenesis or as an accelerator of the process, particularly in specific molecular settings. On the one hand, *Fusobacteria* could be detected in the biofilm covering colonic mucosa of patients with genetically determined FAP and the enrichment of *Fn* occurs already in early-stage carcinoma (Nakatsu et al., 2015). On the other side, the rescue of bacterial DNA, as well as of live bacteria from metastatic lesions, resembles the maintenance of a clonal alteration conferring a selective advantage. Accordingly, treatment with antibacterial drugs inhibited the growth of *Fn^+^* xenografts, suggesting its participation in the maintenance of clonal expansion. In addition, it has been shown that *Fn* can orchestrate the network of Toll-like receptors (TLRs), miRNAs, and ultimately autophagy, contributing to the emergence of chemoresistance. In a future perspective, the confirmation of these data might yield valuable insights to improve CRC clinical management.

In conclusion, although we are far from understanding the involvement of the components of the intestinal microbiome in the development and progression of CRC, *Fn* is paving the way by representing the first exogenous feature contributing to CRC molecular profiling.

## Abbreviations

16S: Subunit 16
5-FU: 5-fluorouracil
APC: Adenomatous Polyposis Coli
BECN1: Beclin 1
BMI: Body Mass Index
BRAF: B-Raf Proto-Oncogene, Serine/Threonine Kinase
CAPOX: Capecitabine plus oxaliplatin
CD3: Cluster of Differentiation 3
CD45RO: or UCHL1, ubiquitin carboxyl terminal esterase L1
CD8: Cluster of Differentiation 8
CHD7: Chromodomain Helicase DNA Binding Protein 7
CHD8: Chromodomain Helicase DNA Binding Protein 8
CIMP: CpG island methylator phenotype
CIN: Chromosomal Instability
CRC: Colorectal cancer
CRIS A–E: CRC intrinsic subtypes A–E
CXCL16: C-X-C Motif Chemokine Ligand 16
EMT: Epithelial-to-Mesenchymal Transition
EPCAM: Epithelial Cell Adhesion Molecule
FadA: Adhesin A
FAP: Familial adenomatous polyposis
Fap2: Fusobacterium autotransporter protein 2
FIT: Faecal Immunochemical Test
Fn: Fusobacterium Nucleatum
FOLFOX: Leucovorin (folinic acid), fluorouracil, plus oxaliplatin
FOXP3: Forkhead Box P3
Gal-GalNAc: D-galactose N-Acetyl-D-galactosamine
HR: Hazard-ratio
IL-6: Interleukin 6
KRAS: KRAS Proto-Oncogene, GTPase
LARC: Locally Advanced Rectal Cancer
LPS: Lipopolysaccharides
MAP1LC3 (LC3): Microtubule Associated Protein 1 Light Chain 3 Alpha
MDSCs: Myeloid-derived suppressor cells
miRNAs: Micro RiboNucleic Acid
MLH1: MutL Homolog 1
MMR: DNA Mismatch Repair
MMRd: DNA mismatch repair deficiency
mRNA: Messenger Ribonucleic Acid
MSH2: MutS Homolog 2
MSH6: MutS Homolog 6
MSI: Microsatellite Instability
MSS: Microsatellite Stable
MUTYH: MutY DNA Glycosylase
MYD88: Myeloid differentiation primary response 88
NF-kB: Nuclear factor-κB
NGS: Next Generation Sequencing
NusG: Transcription termination factor NusG
OR: Odds-ratio
PCR: Polymerase Chain Reaction
PMS2: PMS1 Homolog 2
qPCR: Quantitative Polymerase Chain Reaction
rDNA: Ribosomal Desoxy- (or Deoxy-) riboNucleic Acid
RIG-1: Retinoic acid-inducible gene 1
RNA: RiboNucleic Acid
RNA-ISH: RNA *in situ* hybridization
rpoB: RNA polymerase subunit beta
SQSTM1 (p62): Sequestosome 1
SSAs: Sessile Serrated Adenomas
TIGIT: T Cell Immunoreceptor With Ig And ITIM Domains
TIL: Tumour-infiltrating lymphocyte
TLRs: Toll-like receptors
TLR4: Toll-like receptor 4
TNF: Tumour necrosis factor
TNM: tumour (T), nodes (N), and metastases (M)
TP53: Tumour Protein P53
TWIST1: Twist Family BHLH Transcription Factor 1
Wnt: Wingless-related integration site
